# On the Nature of Improper Hydrogen Bonding in RCH_2_F and RCHF_2_ Motifs

**DOI:** 10.1002/anie.202518500

**Published:** 2025-11-11

**Authors:** Bruno A. Piscelli, Michael Bühl, Rodrigo A. Cormanich, David O'Hagan

**Affiliations:** ^1^ University of Campinas Chemistry Institute Monteiro Lobato Street, Campinas Sao Paulo 13083‐862 Brazil; ^2^ EastChem School of Chemistry University of St Andrews North Haugh St Andrews KY16 9ST UK

**Keywords:** Anion complexation, Difluoromethyl motif, Improper hydrogen bonds, Organofluorine chemistry, Selectively fluorinated alicyclics

## Abstract

The RCH_2_F and RCHF_2_ groups are substituents of interest in medicinal and agrochemicals products. They have a polar aspect relative to the methyl (RCH_3_) or trifluoromethyl (RCF_3_) groups which results in a lowering of Log P's (water affinity). Here we use a computational approach to explore the nature of the interaction between RCH_2_F and RCHF_2_ in methanes and ethanes with chloride ion (and water), as a hydrogen bonding acceptor. A key observation is that the hydrogen atoms geminal to the fluorine(s) become less positively charged with increasing fluorination, a trend anticipated to weaken, not strengthen, their hydrogen bonding interactions. However this study demonstrates a dominating role for the electrostatic interaction of the acceptor with the CF carbons and profiles a shift in negative charge density from hydrogen to the carbon and fluorine(s) as chloride ion (or water) approach. The common occurrence of blue shifts (shortening C─H length) in these ‘improper’ or ‘non‐classical’ hydrogen bonds is also explored and is correlated with the electrostatic interactions between the acceptor and the carbon atoms. These observations are extended to C_3_─C_6_ alicyclic rings containing these motifs and predict particularly strong interactions energies between chloride ion and specifically designed organo‐fluoro alicycles.

## Graphical abstract

The nature of ‘improper’ hydrogen bonds is explored in FnC─H⋯X systems, such as RCF_2_H interaction with chloride and water. It is noted that increased fluorination decreases the positive charge density on H but increases it on C, thus these interactions are dominated by electrostatic attraction to C over H. Such interactions map experimental blue and red shift IR phenomenon.

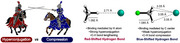

## Introduction

**Figure 1:**
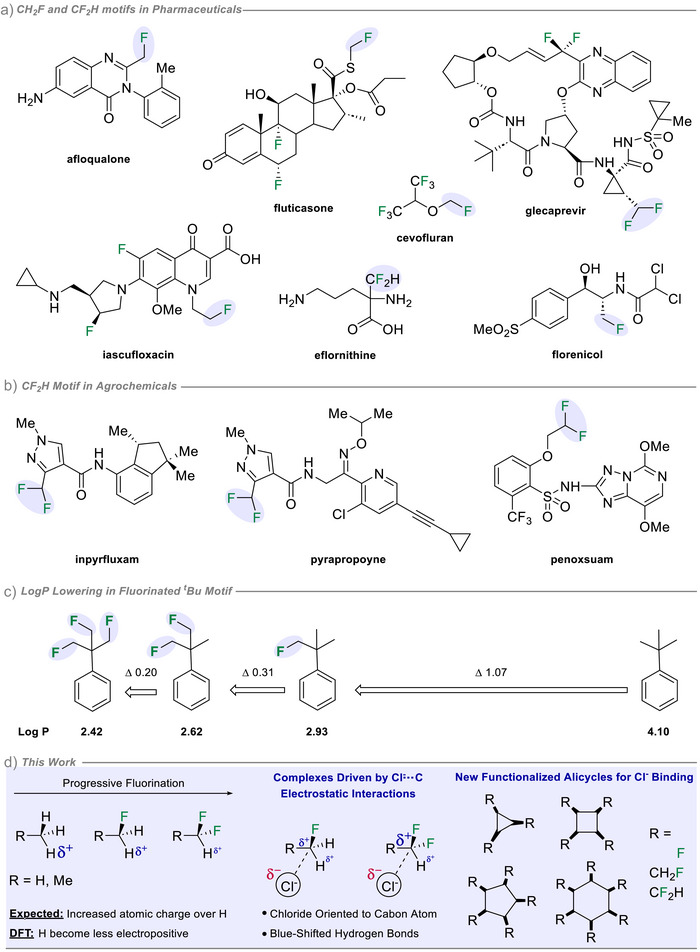
Bioactives containing the ─CH_2_F and ─CHF_2_ groups in a) pharmaceuticals and clinical agents and b) agrochemicals. c) LogP reductions ^[^ ^37^ ^]^ with increasing mono‐fluorinations of the *tert*‐butyl methyl groups. d) This work: DFT analysis reveals that geminal hydrogens become less electropositive with fluorination, and HB acceptors orient toward the carbon center. The study is extended to functionalized alicycles for binding of Cl^−^.

The trifluoromethyl (‐CF_3_) group has been the focus of synthesis attention for many years,^[^ ^1^, ^2^, ^3^ ^]^ however there is a growing interest in lesser fluorinated methyl substituents for medicinal chemistry and new materials, as a future role for the trifluoromethyl (‐CF_3_) group^[^ ^4^, ^5^ ^]^ comes under scrutiny because it may constitute a perfluorocarbon under emerging PFAS regulations.^[^ ^6^ ^]^ Lesser fluorinated methyls are anticipated to generate degradation products in the environment that are less persistent and therefore they are becoming a particular focus of attention.^[^ ^7^, ^8^ ^]^ Monofluoromethyl (‐CH_2_F)^[^ ^9^ ^]^ and difluoromethyl (‐CF_2_H)^[^ ^10^ ^]^ groups offer selectively fluorinated motifs that possess design potential for tuning polarities and lipophilicities of lead compounds in bio‐actives discovery chemistry.^[^ ^11^, ^12^, ^13^, ^14^, ^15^ ^]^ The ─CF_2_H group has been particularly prominent in bioactives development as it is recognized as a hydrogen bond donor and as a candidate surrogate of ─OH, ─NH and ─SH.^[^ ^16^, ^17^, ^18^, ^19^, ^20^, ^21^ ^]^ This prominence is also driven by the development of synthetic methods to obtain such motifs,^[^ ^22^, ^23^, ^24^, ^25^, ^26^, ^27^, ^28^, ^29^, ^30^, ^31^ ^]^ and a variety of structurally diverse drugs^[^ ^32^, ^33^, ^34^ ^]^ and agrochemicals^[^ ^35^, ^36^ ^]^ containing these groups have emerged some of which are illustrated in Figure 1.

Unlike hydrocarbons (with, eg., ─CH_3_ groups) these FCH motifs will engage in hydrogen bonding interactions, although the underlying electrostatic and stereo‐electronic aspects that govern these interactions is different to classical hydrogen bonding. Lippard et al.,^[^ ^11^ ^]^ contrasted the hydrogen bonding ability of the ─CF_2_H group with ─OH in model compounds, exploring X‐ray structures, IR and NMR spectroscopy as well as computation. Their analysis supported RCF_2_H^⋯^O(nitro) hydrogen bonding. Notably IR indicated blue shifted hydrogen bonding, indicative of shortening of the CF_2_─H bond length during the interaction. This contrasts with the more typical red shifts and a lengthening of the X─H bonds observed during the interaction associated with classical hydrogen bonding (eg., X═N, O). Zafrani et al.,^[^ ^16^, ^17^ ^]^ and Zhang et al.,^[^ ^18^ ^]^ have explored the proton acidity of compounds of different subclasses carrying the RCHF_2_ motif, such as ArCF_2_H, AlkylCF_2_H and then RX‐CF_2_H groups where X═O, N, and S. Using the difference in ^1^H‐NMR chemical shifts (Δ*δ*) in CDCl_3_ and DMSO (Abraham's method – *A* values)^[^ ^19^ ^]^ they obtained A values which report on the relative hydrogen bonding donor ability of the geminal hydrogen. A consensus has emerged that the RCF_2_H group is a weaker hydrogen bonding donor than an alcohol or a phenol and that it is much more similar in this capacity to an aniline, an amine or a thiophenol. Although a relatively weak hydrogen bond donor overall, the data indicated that donor strength is dependent on the nature of the functional group directly attached to the carbon atom. The greater A values were associated with the heteroatom ethers RX‐CF_2_H and the lesser values with carbon bound ArCF_2_H, AlkylCF_2_H motifs, the former notably having a more electropositive carbon. When functionalized aryl‐heteroatom ethers (ArX‐CF_2_H) were compared there were strong Hammett correlations with electron withdrawing groups strengthening hydrogen bonding donor capacity.

The ─CH_2_F group^[^ ^38^, ^39^ ^]^ has received less attention as it is a less obvious bio‐isostere for alcohols and thiols. Although fluorination is often associated with increasing lipophilicity, the widely used lipophilicity metric, LogP (partition coefficients of a molecule between octanol and water), reduces with monofluorination of a methyl group, indicating reduced lipophilicity/increased hydrophilicity. This has been relatively widely discussed in the literature, as LogP is an important metric in the design of bioactives in pharmaceuticals and agrochemical development.^[^ ^40^, ^41^, ^42^, ^43^ ^]^ In this context we^[^ ^37^, ^44^ ^]^ recently explored the effect of introducing single fluorines into methyl groups such as in the *tert*‐butylbenzene series illustrated in Figure 1c and observed a very significant lowering of LogP with successive mono‐fluorinations of the *tert‐*butyl motif. It is tempting to suggest that the phenomenon arises from polarization of the geminal hydrogens because of the inductive impact of the fluorine, however computational analysis of fluoromethylene (CHF) hydrogens indicated the atomic charges on the geminal hydrogens become *less positive* with fluorinations rather than more positive.^[^ ^45^, ^46^ ^]^ On this basis these hydrogens should be less able to accommodate hydrogen bonding as they are less electropositive than the hydrocarbon, however it is not the case in practice as the interaction energies of these hydrogen bonds increase with fluorinations. This counter‐intuitive observation stimulated a more comprehensive assessment of the electrostatics of these ‘improper’ hydrogen bonding interactions.

Carbon bound hydrogens constitute ‘improper’ C─H bond donors, in contrast to classical heteroatom X─H donors and they often lead to shorter C─H bonds (blue IR shifts).^[^ ^47^, ^48^, ^49^, ^50^, ^51^, ^52^, ^53^, ^54^ ^]^ This shortening arises because there is an unequal competition between classical, n_(Y)_ → σ*_(C─H)_ hyperconjugation weakening/lengthening the C─H bond versus an electrostatic compression of the C─H bond on approach of the acceptor.^[^ ^47^, ^48^, ^54^ ^]^ This compression is steric as most recently argued by Guerra and Bickelhaupt when explored across a range of blue shifted trifurcated hydrogen bonding systems.^[^ ^54^ ^]^ It has also been argued for example by Weinhold^[^ ^47^ ^]^ to align to Bent's rule ^[^ ^55^ ^]^ where a rehybridization at C induced by electrostatics will also result in C─H bond shortening. In this study we explore improper hydrogen bonding interactions between chloride ion (and water) across mono‐, di‐, and tri‐ fluoromethanes and fluoroethanes. As an outcome we wish to profile the influence of the electrostatic charge on the carbon atom rather than the geminal hydrogens in the CFH systems, as a key driver of these ‘improper’ hydrogen bonds. Our study then extended to exploring chloride interactions with C_3_─C_6_ alicycles containing all‐*cis* ─CH_2_F and CF_2_H motifs attached to each carbon of the rings. Strong complexation interactions are identified with these higher ordered alicycles which confers on them unexpectedly polar properties.

## Results and Discussion

### Fluoromethanes

**Figure 2:**
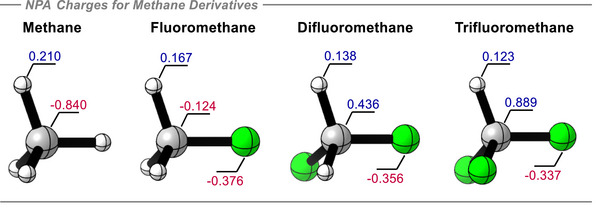
NPA‐calculated atomic charges (+ve in blue, ‐ve in red) for the C, H, and F atoms along the fluoromethane series, at the B3LYP‐D3/def2‐QZVP theoretical level.

In the first instance the interaction energies and atomic charges between chloride ion and progressively fluorinated methanes were explored, by monitoring chloride ion approaching on a trajectory 180° to a C‐H bond. This trajectory was selected as conventional stereo‐electronics suggest that it should form the strongest hydrogen bonding interaction.^[^ ^47^, ^48^ ^]^ It was anticipated that the interactions will become stronger as more fluorines are introduced. This is indeed the case as discussed below. However, it was observed that the NPA‐calculated atomic charge for the hydrogen atoms in methane is +0.210e, and this progressively decreases to +0.167e, +0.138e, and +0.123e respectively for fluoro‐, difluoro‐, and trifluoro‐ methanes, as illustrated in Figure 2. This trend should not support stronger hydrogen bonding because of an expected decreasing electrostatic attraction between H and the acceptor. However, rather than a positive charge accumulating on the hydrogen atoms, instead the positive charge concentrates on carbon. At the extremes, the carbon of methane (CH_4_) is negatively charged (−0.840e) and carries the most electropositive hydrogens of the series whereas the carbon in trifluoromethane (HCF_3_) acquires a large positive charge (+0.889e) and has the least electropositive hydrogen of these methanes. The electrostatic profiles of the mono‐ and di‐ fluoromethane structures fit intermediately and progressively within the series.

**Figure 3:**
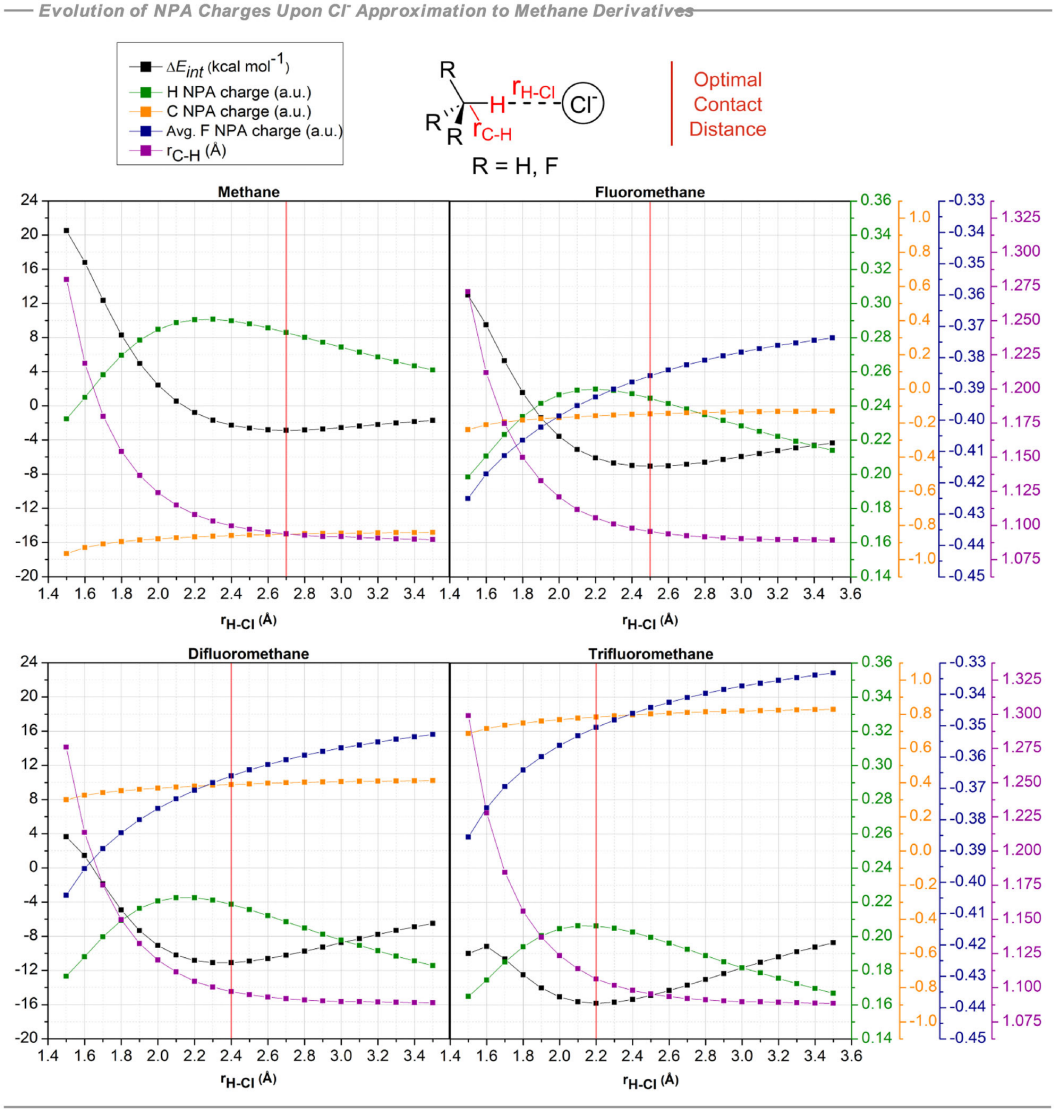
Calculated scans varying C─H⋯Cl^−^ distance in increments of 0.1 Å for methane derivatives. NPA charge changes on interacting H, C, and F atoms, C─H bond length and BSSE‐corrected interaction energies. Calculations at the B3LYP‐D3/def2‐TZVP(D) theoretical level.

An evolution of the interaction energies and atomic charges on the carbon (C), hydrogen (H) and fluorine (F) atoms for methane and the fluoromethanes, was evaluated as the Cl^−^ anion approached along a C─H bond axis. The profiles monitoring the change of atomic charges, the C─H bond lengths and the interaction energies for these systems are illustrated in Figure 3. Relaxed scans were conducted at 0.1 Å increments varying the distance between Cl^−^ and a C─H hydrogen from 1.5 to 3.5 Å. At each 0.1 Å increment, the NPA charges were assessed. The C─H⋯Cl^−^ angle was constrained to 180° throughout the calculations to minimize complication from secondary interactions with neighboring CH bonds, which ultimately lead to bridged structures (see below).

**Figure 4:**
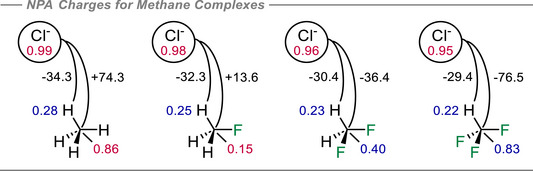
NPA atomic charges (+ve in blue, ‐ve in red) and important NBO electrostatic interactions (in kcal mol^−1^) for the optimal methane interactions with Cl^−^ calculated at the B3LYP‐D3/def2‐QZVP(D) theoretical level.

At the optimal contact distance with chloride ion in each case approaching through a constrained 180° C─H⋯Cl^−^ trajectory, the interaction energies become increasingly stabilizing with increasing fluorination. The trifluoromethane (−15.7 kcal mol^−1^) complex is more stable than that with difluoromethane (−11.1 kcal mol^−1^) and then fluoromethane (−7.1 kcal mol^−1^), with methane displaying the weakest interaction (−2.9 kcal mol^−1^). Notably as chloride ion approaches the hydrogens they become significantly more electropositive. The charge on carbon increases only very slightly as the system approaches the optimal C─H⋯Cl^−^ distance, instead charge density transfers from the hydrogen to the fluorine(s) which get progressively more negative during these interactions. Key interactions are separated out in Figure 4. For example, in methane, the stabilizing C─H⋯Cl^−^ interaction (−34.3 kcal mol^−1^) is offset by a strong electrostatic repulsion between the carbon atom and Cl^−^ (+74.3 kcal mol^−1^). The result overall is a weakly stabilising interaction energy. Upon monofluorination, the hydrogens become less electropositive and the carbon less electronegative, going from −0.86e for the methane C to almost neutral (−0.15e) in fluoromethane. This significantly reduces the destabilizing C⋯Cl^−^ interaction from +74.3 kcal mol^−1^ in methane to +13.6 kcal mol^−1^ in fluoromethane. Notably the C─H⋯Cl^−^ interaction remains almost unchanged at −32.3 kcal mol^−1^, a value which does not change significantly from fluoromethane to trifluoromethane (−29.4 kcal mol^−1^). With the addition of a second fluorine atom on the methane, the carbon now exhibits a positive charge (+0.40e), turning a previously destabilizing C⋯Cl^−^ interaction into a stabilizing one (−36.4 kcal mol^−1^). This interaction becomes even stronger in trifluoromethane (−76.5 kcal mol^−1^) resulting in the strongest methane to Cl^−^ interaction energy of the series. Note that the qualitative charge distributions and the resulting electrostatic arguments developed here are rather insensitive to the particular population analysis or density partitioning scheme employed (see Table S1).

The interaction energies between chloride and the fluoromethanes were further assessed by high‐level wave function calculations (DLPNO‐CCSD(T)/aug‐cc‐pVQZ), and the nature of the interactions was decomposed using the Local Energy Decomposition (LED) approach.^[^ ^56^ ^]^ In LED, the interaction energy of two (or *n*) fragments can be separated into a number of chemically meaningful terms according to Equation (1);

(1) $$\Delta E=\Delta E_{int}+\Delta E_{geo-prep}+E_{BSSE}$$

where the Δ*E_int_* term is the electronic interaction, Δ*E*_*geo* − *prep*_ is the geometric preparation contribution that accounts for the differences between equilibrium molecular geometries of isolated fragments and those in a complex, and *E_BSSE_* which accounts for the Basis Set Superposition Error, calculated according to the Counterpoise method. The electronic interaction energy Δ*E_int_* is further deconvoluted according to the DLPNO‐CCSD(T)‐LED decomposition scheme:

$$\Delta E_{int}=\Delta E_{int}^{HF}+\Delta E_{int}^{C}$$

(2) $$\begin{array}{cc} \Delta E_{int} & =\Delta E_{el-prep}^{HF}+E_{elstat}^{HF}+E_{exch}^{HF}+\Delta E_{non-disp}^{C}\\ & \;+\Delta E_{disp}^{C}+\Delta E_{int}^{C-\left(T\right)} \end{array}$$

The interaction energy Δ*E_int_* is decomposed into the Hartree‐Fock level contribution ($\Delta E_{int}^{HF}$) and corrections due to electronic correlation ($\Delta E_{int}^{C}$). The former is further decomposed into the electronic preparation contribution ($\Delta E_{el-prep}^{HF}$), which corresponds to the energy cost to distort the electronic structure of the isolated fragments into that optimal for complex formation, and also into the attractive electrostatic ($E_{elstat}^{HF}$) and exchange ($E_{exch}^{HF}$) contributions. Additionally, the CCSD correlation term $\Delta E_{int}^{C}$ is partitioned into the London dispersion interaction energy ($\Delta E_{disp}^{C}$) and non‐dispersive correlation contributions ($\Delta E_{non-disp}^{C}$), which provide corrections to the Hartree‐Fock polarization effects, i.e. “dynamic charge polarization.” Finally, the $\Delta E_{int}^{C-(T)}$ provides energy corrections from the perturbative triple excitations (Table 1).

**Table 1:** DLPNO‐CCSD(T)/aug‐cc‐pVQZ/LED//B3LYP‐D3/def2‐QZVP(D) energy terms for methane derivatives interacting with Cl^−^ anion.

	Methane	Fluoromethane	Difluoromethane	Trifluoromethane
Δ*E*_*geo* − *prep*_	0.02	0.32	0.67	1.16
$\Delta E_{el-prep}^{HF}$	13.81	22.05	28.49	37.57
$E_{elstat}^{HF}$	−12.93	−24.22	−34.21	−47.10
$E_{exch}^{HF}$	−2.14	−3.38	−4.28	−5.48
$\Delta E_{non-disp}^{C}$	−0.56	−0.45	−0.16	0.14
$\Delta E_{disp}^{C}$	−1.03	−1.32	−1.58	−1.83
$\Delta E_{int}^{C-(T)}$	−0.27	−0.27	−0.23	−0.19
*E_BSSE_*	0.13	0.22	0.28	0.35
Δ*E* ***_int_***	−2.98	−7.05	−11.01	−15.38

Notably, the interaction energies between the methanes and Cl^−^ anion calculated at the DFT level are in excellent agreement with the DLPNO‐CCSD(T)/aug‐cc‐pVQZ wave function method, which further supports the choice of the functional (B3LYP‐D3) and basis set [def2‐QZVP(D)] for the description of these systems. The geometric preparation term (Δ*E*_*geo* − *prep*_) is destabilizing in all cases and increases in magnitude with further fluorination of the methane systems, reflecting progressively closer C‐H···Cl^−^ contacts from methane to trifluoromethane (Figure 3). However, this term is relatively small and plays a secondary role in the overall interaction energy. The large, positive electronic preparation term ($\Delta E_{el-prep}^{HF}$) is counteracted by attractive contributions from exchange ($E_{exch}^{HF}$) and, primarily, electrostatic interactions ($E_{elstat}^{HF}$), as expected in a hydrogen bonding interaction. Overall, the interaction energy at the Hartree‐Fock level ($\Delta E_{el-prep}^{HF}+E_{elstat}^{HF}+E_{exch}^{HF}$) is stabilizing and accounts for the majority of the interaction energy upon complex formation, clearly indicating that dispersion effects do not have a particularly significant role in these hydrogen bonds. Both dispersive and non‐dispersive energy terms are stabilizing but they are small compared to electrostatics, in agreement with electrostatic‐driven hydrogen bonds.^[^ ^57^ ^]^ Notably, the significance of the electronic correlation terms ($\Delta E_{non-disp}^{C}+\Delta E_{disp}^{C}+\Delta E_{int}^{C-(T)}$) decreases with fluorination. For example, in methane, electronic correlation corrections increase the interaction energy by 138% (from −1.25 to −2.98 kcal mol^−1^). However, this relative increase diminishes in fluoromethane (35%) and further decreases in difluoromethane (18%) and then trifluoromethane (11%), highlighting the increasing electrostatic component for hydrogen bonding upon progressive fluorination.

**Figure 5:**
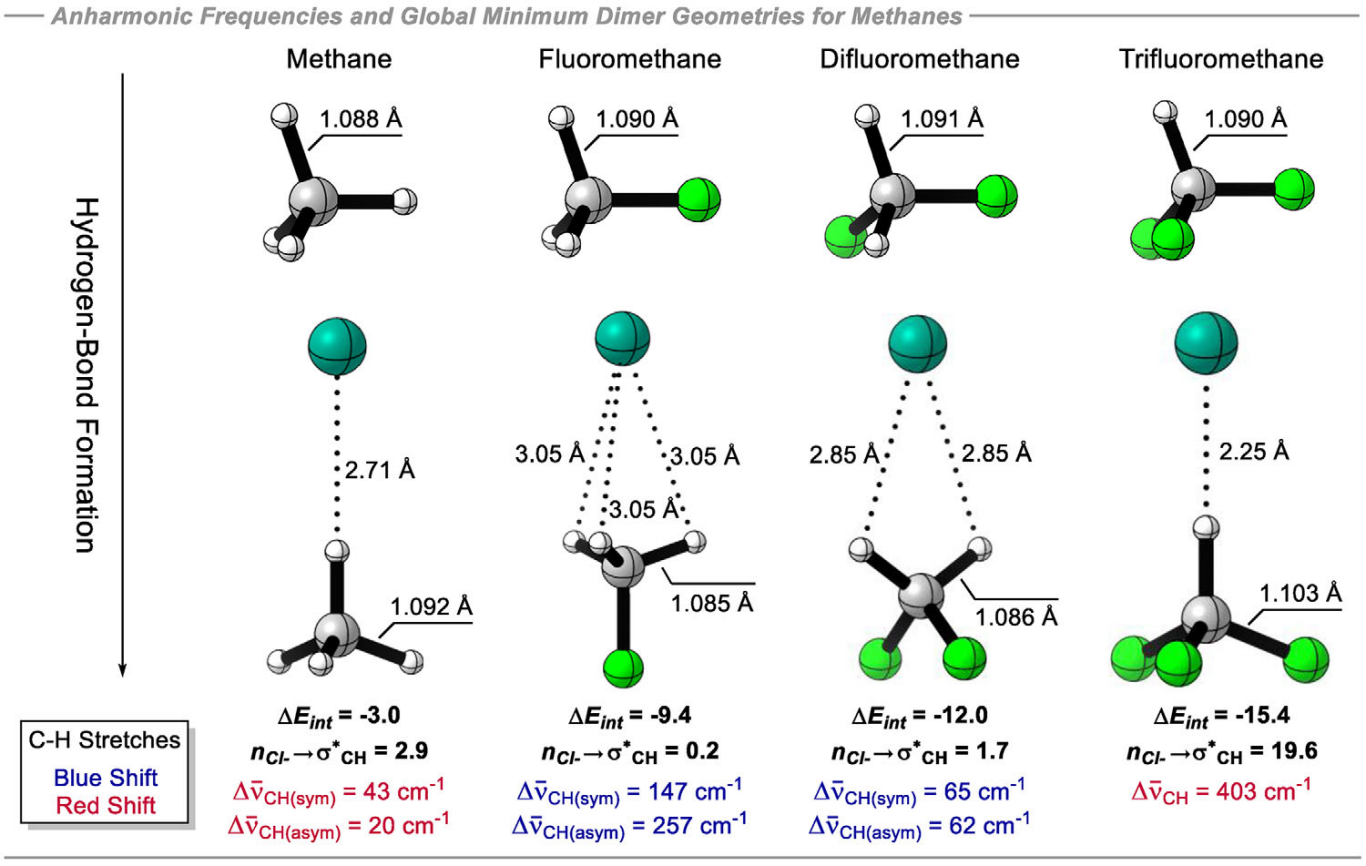
Anharmonic C─H stretch frequencies differences between the non‐complexed (upper) and complexed to Cl^−^ (lower) global minimum geometries of methane derivatives, and NBO hyperconjugative interactions between fragments obtained at the B3LYP‐D3/def2‐QZVP(D). Interaction energies (Δ*E_int_*) at the DLPNO‐CCSD(T)/aug‐cc‐pVQZ, in kcal mol^−1^.

When evaluating the C─H stretching frequencies for both unbound methane derivatives and their global minimum complexes with Cl^−^ (optimized without geometric constraints, Figure 5), an interesting trend emerges. The anion interacts with methane in an almost linear 180° C─H⋯Cl^−^ orientation, yielding a contact energy of –3.0 kcal mol^−1^, virtually identical to the constrained trajectory model (–2.98 kcal mol^−1^). Geometric analysis reveals a slight C─H bond elongation upon binding, increasing from 1.088 Å in the free molecule to 1.092 Å in the complex and resulting in modest red shifts (Δν_CH_) in the vibrational frequencies of 43 cm^−1^ for the symmetric and 20 cm^−1^ for the asymmetric stretch. The 180° alignment is optimal for stereoelectronic transfer of electron density into the σ*_C‐H_ orbital, which amounts to 2.9 kcal mol^−1^ according to NBO analysis and contributes to C─H bond elongation. By contrast, for fluoromethane, the global minimum structure has Cl^−^ interacting directly with the carbon atom, stabilized by three C─H⋯Cl^−^ contacts within a C_3_ᵥ‐symmetrized geometry. This configuration is energetically favored by –2.4 kcal mol^−1^ relative to the 180° constrained hydrogen interaction model. Notably, in this case, the *n*_Cl‐_ → σ*_C‐H_ electron delocalization is very weak (0.2 kcal mol^−1^) and the C─H bonds actually shorten by 0.005 Å, leading to substantial blue shifts of 147 cm^−1^ (symmetric) and 257 cm^−1^ (asymmetric) in the vibrational stretches. This blue‐shift in fluoromethane was also recently reported by Guerra and Bickelhaupt et al. in a broader study of trifurcated hydrogen bonding, recently named “tetrel bonds”,^[^ ^54^ ^]^ where steric compression upon backside approach by Cl^−^ was concluded to play the predominant role in C─H shortening. Similar tetrel interactions involving the ─CH_2_F motif have also been reported by Lectka et al.^[^ ^58^ ^]^ Accordingly, NBO analysis also revealed a strong steric contact between Cl^−^ and the C─H bonds in this case, as well as a rehybridization at carbon upon Cl^−^ binding, in agreement with previous findings^[^ ^47^, ^48^ ^]^ (see ESI for further details). A similar behavior is observed in difluoromethane, where Cl^−^ preferentially interacts with the carbon atom while simultaneously engaging both hydrogens, resulting in a stronger interaction energy of –12.0 kcal mol^−1^, compared to –11.0 kcal mol^−1^ for the constrained 180° contact. Here, the C─H bonds shorten slightly by 0.004 Å, accompanied by moderate blue shifts of 65 cm^−1^ (symmetric) and 62 cm^−1^ (asymmetric) in the IR stretches. Interestingly, although compression dominates, in this case *n*_Cl‐_ → σ*_C‐H_ hyperconjugation is considerably stronger (1.7 kcal mol^−1^) than that in fluoromethane, which contributes to C─H bond weakening and this explains the smaller calculated blue shifts relative to those in fluoromethane. These values align with the blue shifts reported by Lippard et al.,^[^ ^11^ ^]^ for CF_2_H⋯O(nitro) hydrogen bonds. In contrast to the mono‐ and di‐ fluoromethane, trifluoromethane displays a red shift on complexation. Unconstrained chloride ion adopts a linear 180° C─H⋯Cl^−^ trajectory toward the hydrogen to minimise repulsion from the fluorines. This alignment, which is dictated by electrostatics, none‐the‐less maximises electron density transfer into the σ*_CH_ orbital (19.6 kcal mol^−1^) leading to an overall C─H bond elongation of 0.013 Å and a pronounced red shift of 403 cm^−1^ in the C─H stretching frequency. A similar observation regarding the role of the electropositive C in trifluoromethane in stabilizing this red shift interaction, was recently noted by Paul too.^[^ ^59^ ^]^

### Fluoroethanes

**Figure 6:**
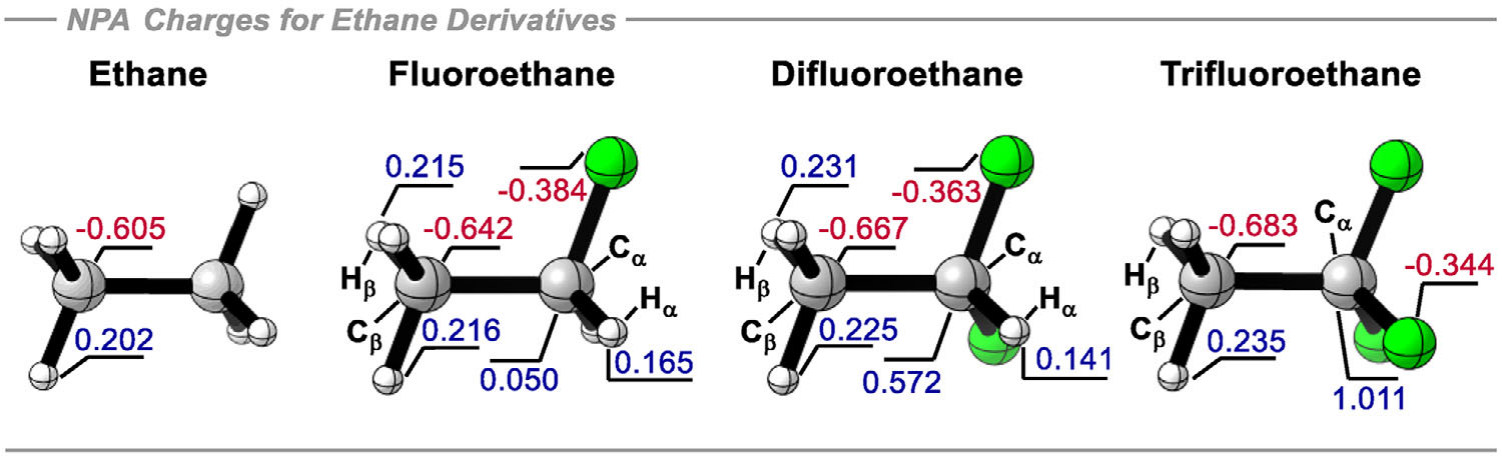
NPA‐calculated atomic charges (+ve in blue, ‐ve in red) for the C, H, and F atoms for ethane and its fluorinated derivatives at the B3LYP‐D3/def2‐QZVP theoretical level.

Our study is now extended to an analysis of ethane and its mono‐, di‐, and trifluorinated analogues, with fluorine atoms introduced progressively to the same carbon atom. Following the trend observed in the methanes, progressive fluorination decreases the atomic charge on the geminal hydrogen atoms, however the vicinal hydrogens become more positively charged, as illustrated in Figure 6. In ethane, all hydrogens are electropositive (+0.202e), while both carbons are negatively charged (−0.605e). Upon mono‐fluorination, the carbon directly attached to the fluorine atom (hereafter referred to as the α carbon) assumes a slight positive charge (+0.050e), and the two geminal hydrogens become less electropositive (+0.165e). Conversely, the carbon in the methyl group (the β carbon) becomes more negatively charged (−0.642e), and the three vicinal hydrogens become more electropositive (+0.216e). This trend continues in difluoro‐ and trifluoroethane, with the α carbon in trifluoroethane reaching a full positive charge (+1.011e) and the vicinal hydrogens becoming significantly more electropositive, with an atomic charge of +0.235e.

**Figure 7:**
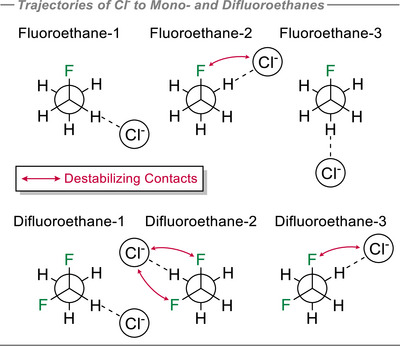
The three trajectories explored for Cl^−^ approaching fluoroethane (top) and difluoroethane (bottom). Important F↔Cl^−^ electrostatic clashes are illustrated by red arrows.

**Figure 8:**
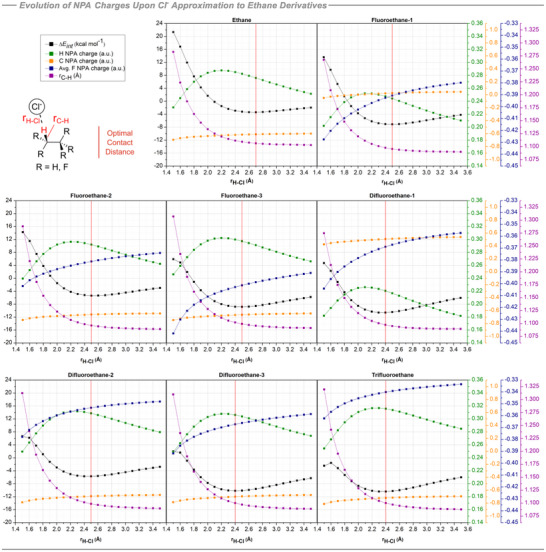
Calculated scans varying C─H⋯Cl^−^ distance at increments of 0.1 Å for ethane derivatives. NPA charges on interacting H and C, average NPA charges on F atoms, C─H bond length and BSSE‐corrected interaction energies. Calculations at the B3LYP‐D3/def2‐TZVP(D) theoretical level.

As with the methane analogues, the distance between Cl^−^ and one of the hydrogen atoms in each ethane analogue was scanned from 1.5 to 3.5 Å at 0.1 Å increments imposing linear C─H⋯Cl^−^ arrangements. If the model is confined to the staggered conformation as illustrated in Figure 7, fluoroethane and difluoroethane each have three chemically distinct hydrogen atoms, leading to three possible interactions with chloride anion along C─H trajectories. These interactions were explored, and the profiles for each scenario are depicted in Figure 8.

The interaction energy for ethane itself is relatively small, at −3.4 kcal/mol. However, a notable trend emerges for monofluorinated ethane. In fluoroethane‐1, where Cl^−^ interacts with an α‐hydrogen in the ─CH_2_F moiety, the interaction energy is −7.1 kcal mol^−1^. This is weaker than the interaction energy where Cl^−^ interacts with a β‐hydrogen, anti‐periplanar to the fluorine atom (−8.8 kcal mol^−1^) in fluoroethane‐3. In contrast, the interaction energy is weakest overall (−5.3 kcal mol^−1^) when the contact is to one of the *gauche* β‐hydrogens, as represented in the fluoroethane‐2 structure. The energy difference between the fluoroethane‐2 and fluoroethane‐3 systems is consistent with electrostatic repulsion between Cl^−^ and the fluorine atoms in the *gauche* relative to the anti‐periplanar arrangement.

The interaction energy of chloride with 1,1‐difluoroethane increased relative to monofluoroethane despite having the least positive α‐hydrogen (+0.141e) of the series. Also the strongest interaction with chloride is observed orientated toward this least electropositive hydrogen. For example, in the difluoroethane‐1 system, monitoring the Cl^−^ interaction along the C─H trajectory of the ─CHF_2_ group, the interaction energy is optimal at −10.6 kcal mol^−1^. The interaction energy in difluoroethane‐3, where Cl^−^ interacts with the more electropositive β‐hydrogen (+0.225e) situated antiperiplanar and *gauche* to the fluorines of the CHF_2_ group, is slightly lower at −10.2 kcal mol^−1^. The interaction energy is weakest at −5.7 kcal mol^−1^ in difluoroethane‐2 where Cl^−^ approaches the *most* electropositive β‐hydrogen (+0.231e) sitting *gauche/gauche* to both of the fluorines, but with F↔Cl^−^ repulsions weakening the interaction.

Finally, the interaction energy of chloride anion with 1,1,1‐trifluoroethane was investigated. Due to the symmetry established by the ─CF_3_ group, the chloride interaction was assessed only to the β‐hydrogen (+0.235e) of the staggered conformation of trifluoroethane, and this was evaluated with an optimal stabilization of −10.4 kcal mol^−1^. This interaction energy is similar in magnitude to both the α‐ and antiperiplanar β−hydrogen interactions in difluoroethane. There appears also to be charge transfer from hydrogens to the fluorines as hydrogen bonding becomes optimal.

The interaction energies in the ethane derivatives were also evaluated using the DLPNO‐CCSD(T)/aug‐cc‐pVQZ wave function method and decomposed using the LED scheme, yielding results that closely match those predicted by DFT. The outcomes are consistent with those observed for the methane analogues. The geometric preparation term (Δ*E*_*geo* − *prep*_) is consistently destabilizing but small; the interaction energies at the Hartree‐Fock level ($E_{el-prep}^{HF}+E_{elstat}^{HF}+E_{exch}^{HF}$) are stabilizing in all cases, predominantly driven by the electrostatic component ($E_{elstat}^{HF}$), and the electronic correlation components ($\Delta E_{non-disp}^{C}+\Delta E_{disp}^{C}+\Delta E_{int}^{C-(T)}$) further stabilize the interactions, though their relative importance decreases as the absolute interaction energies increase (Table 2).

**Table 2:** DLPNO‐CCSD(T)/aug‐cc‐pVQZ/LED//B3LYP‐D3/def2‐QZVP(D) energy terms for ethane derivatives interacting with Cl^−^ anion.

	Ethane	Fluoroethane‐1	Fluoroethane‐2	Fluoroethane‐3
Δ*E*_*geo* − *prep*_	0.04	0.33	0.64	0.44
$\Delta E_{el-prep}^{HF}$	14.46	22.83	18.13	23.17
$E_{elstat}^{HF}$	−13.87	−24.77	−18.87	−26.57
$E_{exch}^{HF}$	−2.20	−3.46	−2.78	−3.55
$\Delta E_{non-disp}^{C}$	−0.61	−0.48	−0.61	−0.58
$\Delta E_{disp}^{C}$	−1.17	−1.54	−1.35	−1.53
$\Delta E_{int}^{C-(T)}$	−0.31	−0.32	−0.33	−0.34
*E_BSSE_*	0.18	0.25	0.21	0.24
Δ*E* ***_int_***	−3.49	−7.17	−4.96	−8.74

	Difluoroethane‐1	Difluoroethane‐2	Difluoroethane‐3	Trifluoroethane
Δ*E*_*geo* − *prep*_	0.64	0.54	0.97	0.74
$\Delta E_{el-prep}^{HF}$	29.33	22.96	29.06	29.32
$E_{elstat}^{HF}$	−34.18	−23.01	−32.81	−33.28
$E_{exch}^{HF}$	−4.37	−3.53	−4.46	−4.49
$\Delta E_{non-disp}^{C}$	−0.24	−0.79	−0.68	−0.69
$\Delta E_{disp}^{C}$	−1.78	−1.54	−1.72	−1.69
$\Delta E_{int}^{C-(T)}$	−0.29	−0.38	−0.37	−0.36
*E_BSSE_*	0.31	0.25	0.28	0.30
Δ*E* ***_int_***	−10.58	−5.51	−9.71	−10.15

**Figure 9:**
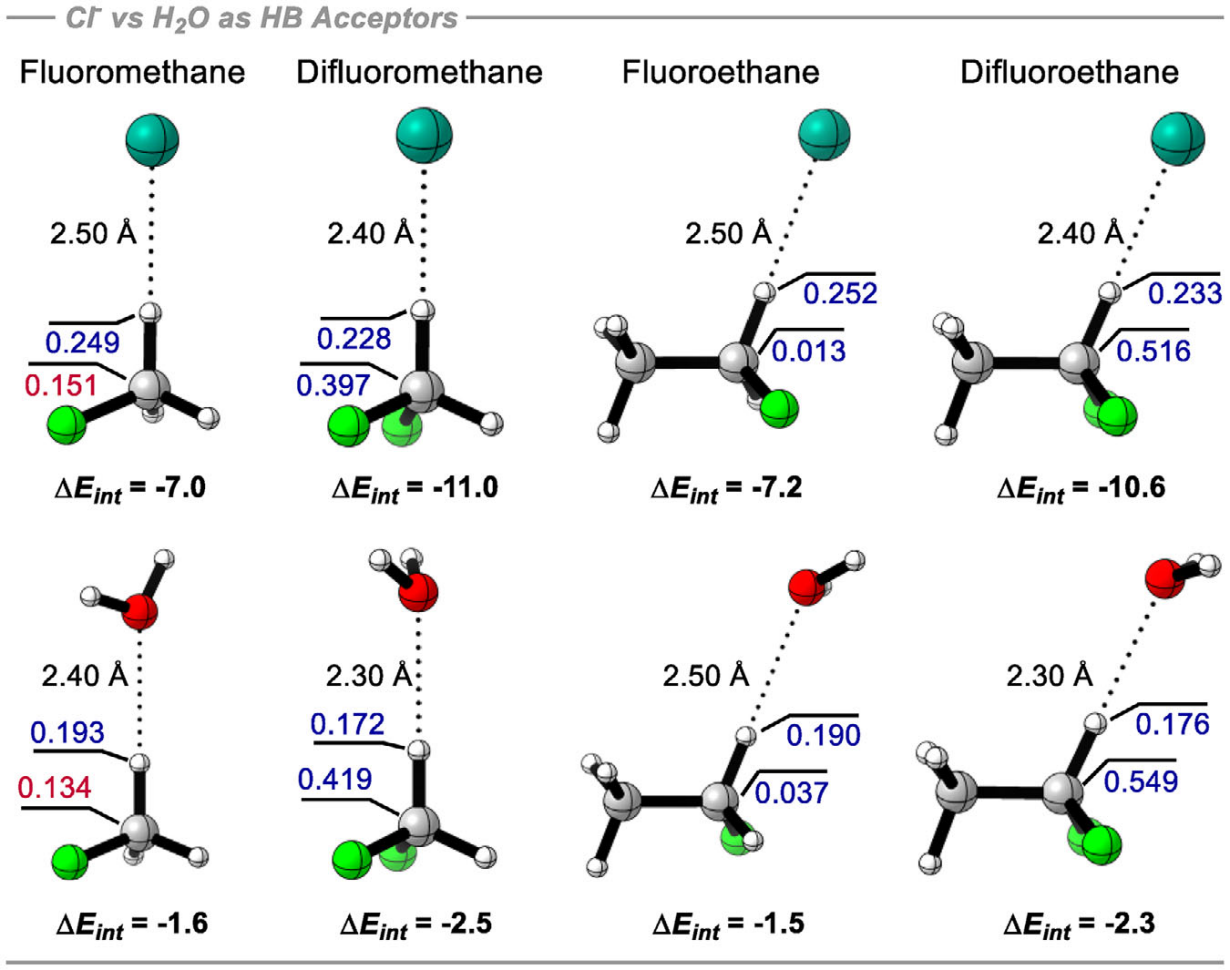
Comparison between the hydrogen bonds of mono‐ and difluoro methane and ethane with Cl^−^ *vs* H_2_O on the 180° constrained trajectories. NPA charges (+ve in blue, ‐ve in red) and geometries obtained at the B3LYP‐D3/def2‐QZVP(D) theoretical level, and interaction energies (Δ*E_int_*) at the DLPNO‐CCSD(T)/aug‐cc‐pVQZ theoretical level in kcal mol^−1^.

In order to simulate a more realistic scenario pertinent to understanding the nature of these motifs in the development of bioactive compounds, similar calculations were conducted using H_2_O as the HB acceptor instead of Cl^−^ (Figure 9). The general trends observed with water follow those observed with chloride anion, although the magnitude of each interaction is smaller, consistent with a neutral HB acceptor. For instance, the interaction of Cl^−^ with fluoromethane along the 180° constrained trajectory results in an interaction energy of −7.0 kcal mol^−1^. Replacing Cl^−^ with water as the hydrogen bond acceptor leads to a substantial decrease in the interaction energy to −1.6 kcal mol^−1^. This reduction reflects the markedly different polarization capabilities of the two acceptors. The electron‐rich Cl^−^ induces a significant positive charge on the hydrogen atom (+0.249e), whereas water, being neutral and smaller (2*p* electrons instead of 3*p*), yields a considerably lower polarization (+0.193e), thus weakening the interaction. A similar trend is observed for difluoroethane, where Cl^−^ induces charges of +0.228e on hydrogen and +0.397e on carbon, corresponding to a strong interaction energy of −11.0 kcal mol^−1^. In contrast, with water as the acceptor, the hydrogen polarization drops to +0.172e, and the interaction energy decreases to −2.5 kcal·mol^−1^. This behavior is also observed for mono‐ and difluoroethane, where the interaction energies with Cl^−^ of −7.2 and −10.6 kcal mol^−1^ for the CFH_2_ and CF_2_H motifs, respectively, are significantly reduced to −1.5 and −2.3 kcal·mol^−1^ when water is the HB acceptor. These are constrained trajectories for water. A key distinction between the Cl^−^ and H_2_O systems lies in the amphoteric behavior of water, which can act as both a HB donor and acceptor and consequently gives rise to complexation geometries featuring simultaneous C─H⋯OH_2_ and C─F⋯H─OH hydrogen bonds. Such cooperative interactions significantly strengthen the overall binding, yielding interaction energies as favorable as ∼4.0 kcal mol^−1^ across this series (see Figure S5).

**Figure 10:**
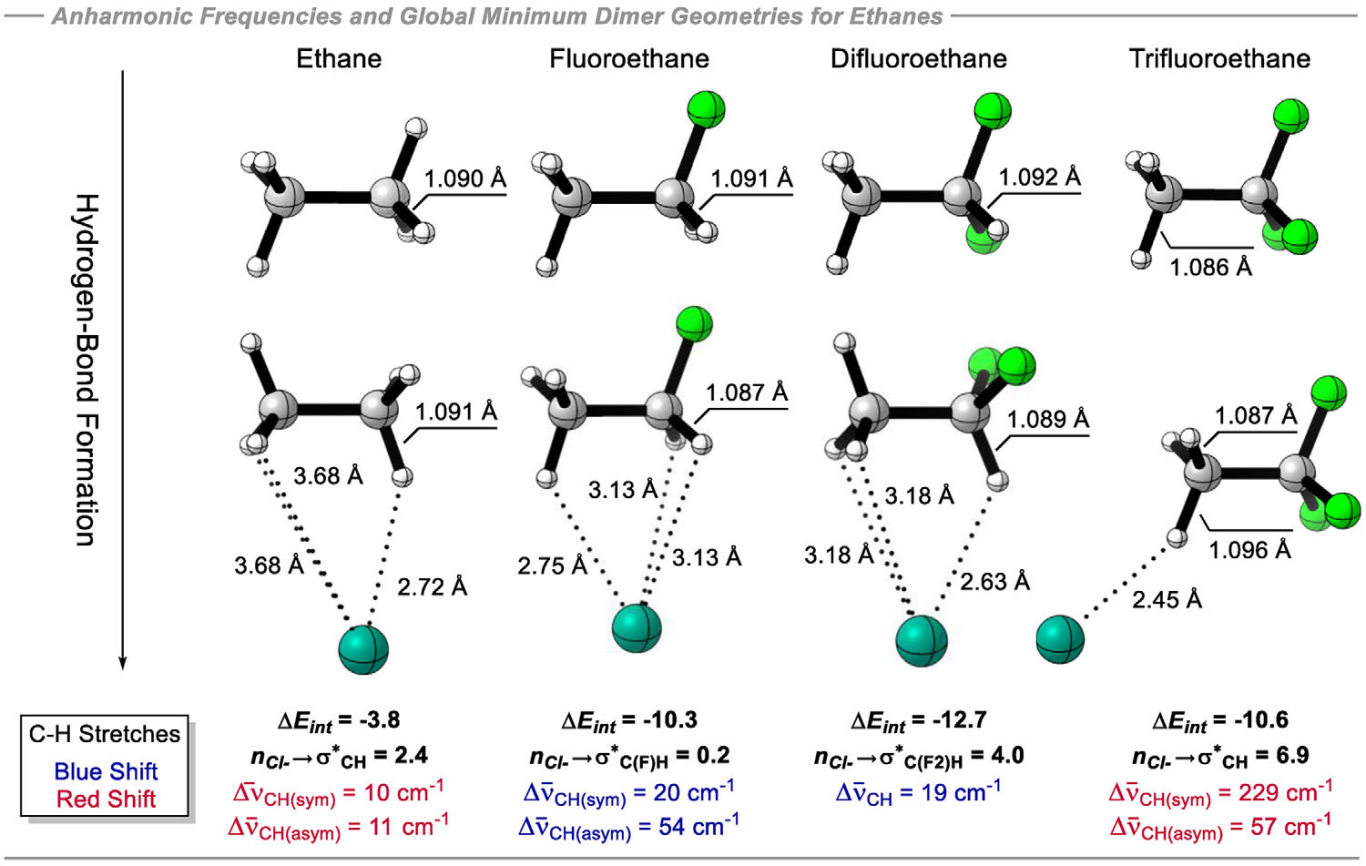
Anharmonic C─H stretch frequencies differences between the non‐complexed (top) and complexed to Cl^−^ (bottom) global minimum geometries of ethane derivatives and NBO hyperconjugative interaction between fragments obtained at the B3LYP‐D3/def2‐QZVP(D) theoretical level. Interaction energies (Δ*E_int_*) at DLPNO‐CCSD(T)/aug‐cc‐pVQZ, in kcal mol^−1^.

A similar IR frequency analysis performed for the methane analogs was extended to the ethanes, revealing a noteworthy trend (Figure 10). In ethane, the chloride interaction is supported only by the electropositive hydrogens, not the electronegative carbons. Electron delocalization from Cl^−^ to σ*_C‐H_ is of moderate strength (2.0 kcal mol^−1^) and causes a C─H bond elongation of only 0.001 Å, resulting in small red shifts of –10 cm^−1^ and –11 cm^−1^ for the symmetric and asymmetric stretching modes, respectively. Upon monofluorination, chloride is attracted to the electropositive C_α_ atom. As Cl^−^ approaches, the C_α_⋯Cl^−^ electrostatic interaction induces a C_α_─H bond shortening of 0.004 Å with a weak *n_Cl‐_* → σ*_C‐H_ interaction (0.2 kcal mol^−1^), accompanied by blue shifts of +20 cm^−1^ (symmetric stretch) and +54 cm^−1^ (asymmetric stretch). A similar C_α_⋯Cl^−^ interaction is observed in difluoroethane, where C─H compression is partially countered by a stronger *n_Cl‐_* → σ*_C‐H_ electron delocalization (4.0 kcal mol^−1^). This results in an overall 0.003 Å shortening of the CF_2_─H bond and a corresponding blue shift of +19 cm^−1^. For trifluoromethane, despite C_α_ being the most electropositive in the series, Cl^−^ preferentially approaches C_β_, avoiding unfavorable F↔Cl^−^ interactions and picking up the electropositive H_β_ hydrogens. Since C_β_ is electron‐rich and accumulates negative charge, Cl^−^ orients to H_β_ (not C_β_), maximizing donation into the σ*_C‐H_ orbital (6.9 kcal mol^−1^). This leads to a significant lengthening (0.010 Å) of the C_β_─H_β_ bond while the other two C_β_─H_β_ bonds experience lesser elongations of 0.001 Å. These structural changes manifest as large red‐shifts of –229 and −57 cm^−1^ for the symmetric and asymmetric stretches, respectively. Thus, comparing methane and ethane outcomes, we can conclude that when the interaction with Cl^−^ is directed to the carbon atoms (mono‐ and difluoromethane, mono‐ and difluoroethane), the C─H bonds tend to become compressed^[^ ^54^, ^60^ ^]^ and they experience blue shifts in the IR spectrum. On the other hand, when the interaction is forced along a 180° trajectory, as in CF_3_H and CF_3_CH_3_, *n_Cl‐_* → σ*_C‐H_ hyperconjugative interactions compensate for the C─H bond compression and this results in elongation of C─H bond and consequently IR red shifts occur.^[^ ^53^, ^54^ ^]^

### Properties of ─CH_2_F and ─CF_2_H Containing Alicyclic Rings

**Figure 11:**
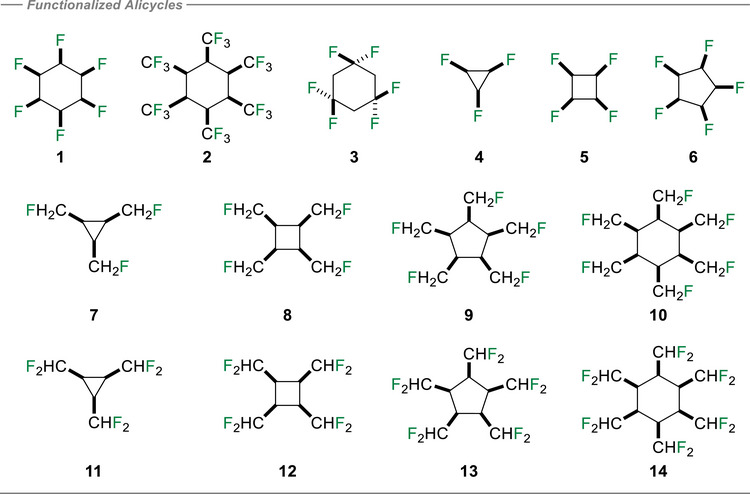
Structures of the selectively fluorinated or all‐*cis* vicinal ─CH_2_F and ─CF_2_H containing alicyclic rings **1‐14**, explored for their chloride ion binding affinities.

Extending our interest in the properties of partially fluorinated alicycles,^[^ ^57^, ^61^, ^62^ ^]^ we now progressed to investigate ─CH_2_F and ─CHF_2_ motifs arranged on alicyclic ring frameworks, to explore if they could cooperatively arrange to display a significant binding affinity to an anion, using chloride ion as a model. In the candidate structures **7**‐**14** in Figure 11, the fluoromethyl motifs are arranged with an all‐*cis* stereochemistry on the ring framework progressing from cyclopropane to cyclohexane. In each case the relative interaction energies with chloride ion, to both the top and bottom faces of these rings systems were calculated, and a particular focus was placed on evaluating the atomic charges at C and H. At the outset the cyclohexanes *cis*‐1,2,3,4,5,6‐hexafluorocyclohexane **1**,^[^ ^62^ ^]^ and all‐*cis* hexakis(trifluoromethyl)cyclohexane **2** ^[^ ^63^ ^]^ were evaluated too to provide a frame of reference as they are experimentally ^[^ ^57^, ^63^ ^]^ demonstrated to show good affinities to chloride ion, and the former also explored in several theory studies.^[^ ^64^, ^65^ ^]^ In these cases chloride associates with the hydrogen faces of the cyclohexanes when they adopt their most stable chair conformations. 1,1,3,3,5,5‐hexafluorocyclohexane **3** was selected too as a new variant in this study.

**Figure 12:**
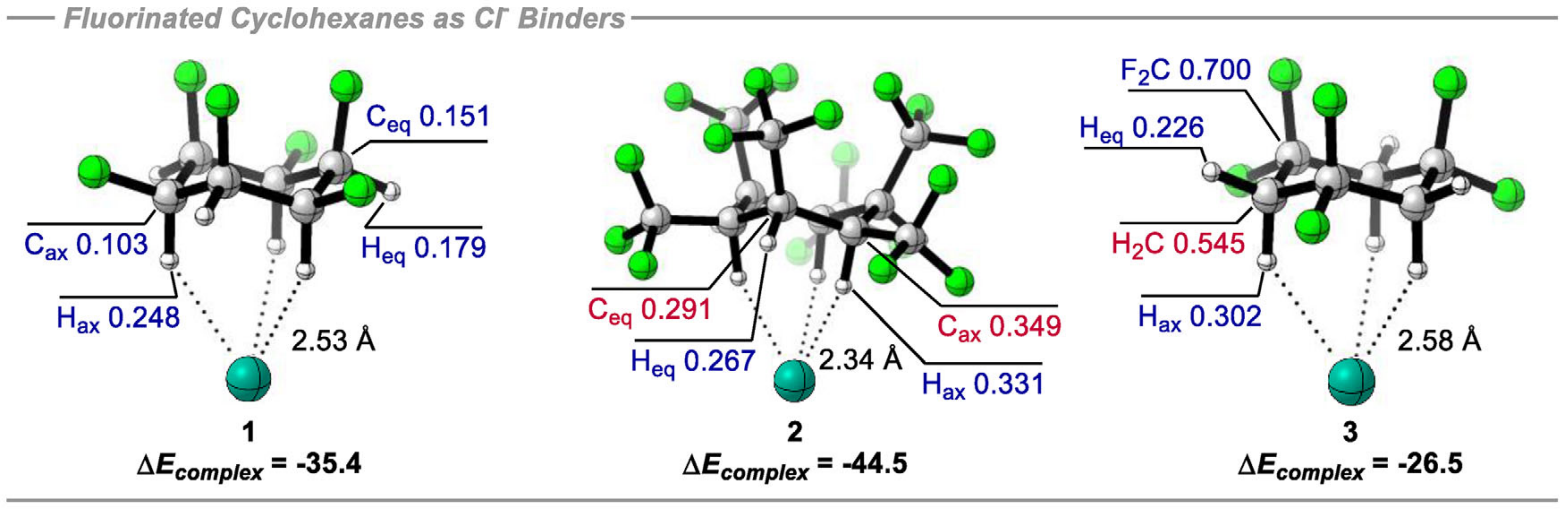
Complexation energies (kcal mol^−1^) and NPA atomic charges (+ve in blue, ‐ve in red) for complexes between cyclohexanes **1**‐**3** and chloride ion (Cl^−^) computed at the B3LYP‐D3/def2‐TZVP(def2‐TZVPD on Cl^−^) theory level and corrected for BSSE using the Counterpoise method.

The calculated binding energies for chloride ion interactions with cyclohexanes **1**‐**3** are summarized in Figure 12. A strong chloride anion complexation to the hydrogen face of all‐*cis*‐1,2,3,4,5,6‐hexafluorocyclohexane **1** was evident, evaluated at the B3LYP‐D3/def2‐TZVP(D) theory level, yielding a binding energy of −35.4 kcal mol^−1^. This is consistent with previous theory studies which variously evaluated binding interactions of **1** and chloride ion at 37.2,^[^ ^57^ ^]^ 35.5,^[^ ^64^ ^]^ and 35.1 kcal mol^−1^.^[^ ^65^ ^]^ Strikingly, hexakis‐(trifluoromethyl)cyclohexane **2** exhibited a significantly higher complexation energy with Cl^−^ at −44.5 kcal mol^−1^. An analysis of the relative atomic charge distributions in **1** and **2** revealed that both the axial hydrogens and the carbon atoms bound to the axial hydrogens, here termed C_ax_, are significantly more electropositive in **2** when compared to **1**. In cyclohexane **1** the carbons all have a modest positive charge and notably those holding the axial hydrogens are actually *less* positive than those bonded to the equatorial hydrogens (C_ax_ +0.103e versus C_eq_ +0.151e). It is the axial hydrogens and their carbons that come into closest contact with the anion and therefore it would appear that the driving force for the interaction in **1** is more associated with the axial hydrogen atoms which carry a charge of +0.248e, and less associated with the carbon atoms which carry a lesser positive charge. By contrast, even though the ring carbon atoms adopt a negative charge (C_ax_ −0.349e, C_eq_ −0.291e) in cyclohexane **2**, the ─CF_3_ groups strongly polarize the axial hydrogens (H_ax_ +0.331e), much more effectively than the fluorines do in **1**, compensating the stronger C↔Cl^−^ contacts and accounting for the much higher binding energy. Cyclohexane **3** makes an interesting contrast with a weaker interaction energy of −26.5 kcal mol^−1^. The alternating ─CH_2_‐ and ─CF_2_‐ groups in the ring dictate that the carbon atoms are either positively (CF_2_ +0.700e) or negatively (CH_2_ −0.545e) charged. It is poignant that although the axial hydrogens carry a significant positive charge (H_ax_ + 0.302), greater than that found in **1**, they are attached to more negatively charged carbons compared to **2** and this weakens the interaction such that it is the weakest of the three, a result of stronger C↔Cl^−^ electrostatic repulsions.

### Alicyclic ‘Nested’ Compounds

**Figure 13:**
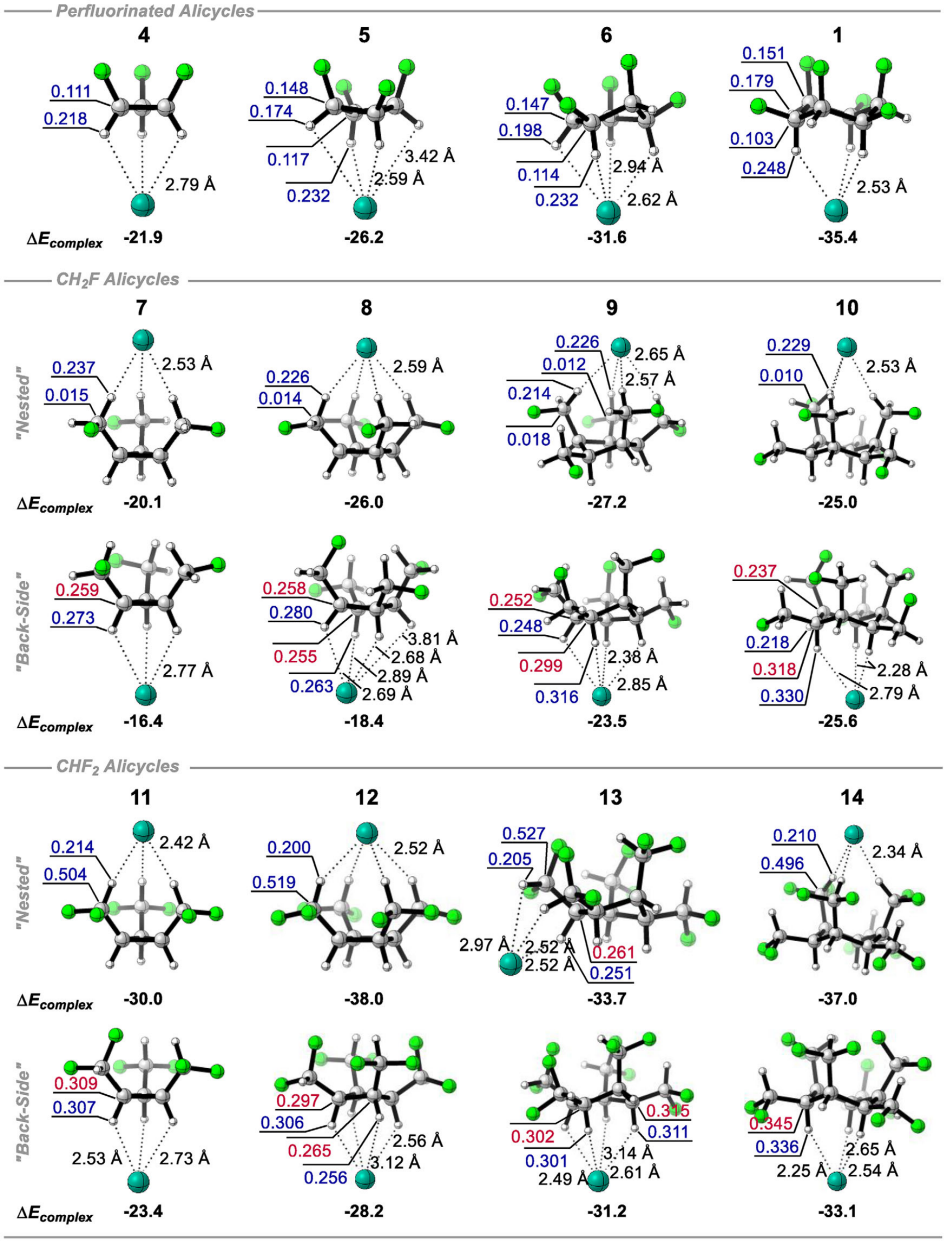
Chloride ion complexation energies (kcal mol^−1^) and NPA atomic charges (+ve in blue, ‐ve in red) calculated at the B3LYP‐D3/def2‐TZVP(D) theory level for alicyclic rings **1** and **4** – **14**.

Motivated by the electrostatic interactions between the methanes, ethanes and alicyclics **1**‐**3**, we now explore C_3_ to C_6_ alicyclic systems **4** – **14**. These ring systems are substituted with ─F, ─CH_2_F, or ─CHF_2_ groups on each ring carbon and with an all‐*cis* configuration in each case, generating ‘nested’ type arrangements. It is already established that chloride ion approaches the hydrogens on the “back side” of cyclohexane **1** away from the triaxial fluorines **1**. The resultant data is summarized in Figure 13.

For the all‐*syn* vicinally fluoro‐substituted rings **1**, **4**, **5** and **6** binding is only to the hydrogen face, with the strongest interaction to cyclohexane **1**. For the other ring systems **7** ‐ **14** both front and backside complexations with chloride ion become reasonable, however for these fluoromethyl appended rings, the ‘back side’ interaction energy between Cl^−^ and the ring bound hydrogens is generally less stable than interactions where complexation is to the ─CH_2_F and ─CHF_2_ groups. An exception is found in the 6‐membered system **10** substituted with ─CH_2_F, where both modes of complexation are essentially isoenergetic, (−25.0 and −25.6 kcal mol^−1^). Across all of these systems, the reference compound all‐*cis*‐1,2,3,4,5,6‐hexafluorocyclohexane **1** remains among the most effective for interaction with Cl^−^, with a complexation energy of −35.4 kcal mol^−1^. This is however surpassed by the ─CHF_2_ containing 4‐ and 6‐ membered rings **12** and **14**, which exhibit complexation energies of −38.0 and −37.0 kcal mol^−1^, respectively. Notably similar interactions to the analogous ─CH_2_F containing 4‐ and 6‐ membered rings **8** and **10** are significantly weaker (−26.0 and −25.0 kcal mol^−1^) despite the ─CH_2_F group possessing the more electropositive geminal hydrogens (+0.229e and +0.229e for **8** and **10** versus +0.200e and +0.210e for **12** and **14** respectively). The better complexation of chloride to ─CHF_2_ over the ─CH_2_F groups is more easily rationalized by the more electropositive C atoms of the ─CHF_2_ groups (+0.014e and +0.010e for **8** and **10** versus +0.519e and +0.496e for **12** and **14** respectively). The ─CF_2_H carbons in rings **11** – **14** are all carrying approximately half a positive charge (∼ +0.50e) whereas the ─CH_2_F carbons in rings **7** ‐ **10** are essentially neutral (∼ +0.01e) and this leads to a weakening of the interaction in the latter case.

A broad potential application of these new alicyclic compounds lies in their use as anion binders in solution for organocatalysis, a role currently dominated by ureas, thioureas, and squaramides.^[^ ^66^, ^67^, ^68^ ^]^ To explore this comparison, we computed the complexation energies of alicycles **1–14** with chloride, including implicit solvation in acetonitrile (Figure S6), and compared the results with that of urea as a reference. Notably, alicycles **2** and **14** exhibited particularly strong binding in this polar medium, with complexation energies of −5.8 and −6.7 kcal mol^−1^, respectively, comparable to that of urea (−5.4 kcal mol^−1^) under the same conditions. These results point to potential application of suitably designed alicyclic of this class as anion binders similar to ureas.

## Conclusion

In summary we have demonstrated that alicyclic hydrocarbons containing ─CH_2_F and ─CHF_2_ motifs display ‘improper’ blue shift hydrogen bonding phenomenon driven by the electropositive nature of the carbon atom directly bonded to fluorine. Ground state charge distributions are such that the geminal ─CHF_n_ hydrogens become increasingly less positive and the carbons increasingly more positive, as fluorines are added. The electrostatic attraction between the acceptor and the ─CF carbon atom is the more significant driver in determining the optimal trajectory and interaction energies. Our study reproduces red and blue shift hydrogen bonds consistent with previous studies^[^ ^45^, ^46^, ^47^ ^]^ which have invoked a balance between C─H bond compression and n → σ*_(C─H)_ hyperconjugation. However recognition of the importance of the electropositive nature of the carbon in these systems offers a perspective that should better enable the design of future performance molecules and materials based on these partially fluorinated substituents.

## Conflict of Interests

The authors declare no conflict of interest.

## Supporting information



Supporting Information

## Data Availability

Additional computational details and supporting results are available free of charge at the Electronic Supporting Information (ESI). The research data supporting this publication can be accessed from Data underpinning “On the Nature of Improper Hydrogen Bonding in RCH_2_F and RCHF_2_ Motifs”, University of St Andrews Research Portal, https://doi.org/10.17630/2b2156d9-df59-466a-8863-14dd497e3a11.
