# Supplementary material for: On the Nature of Improper Hydrogen Bonding in RCH2F and RCHF2 Motifs
Source: Angew Chem Int Ed Engl. 2025 Nov 11;65(2):e18500. doi: 10.1002/anie.202518500 (PMC12790382; doi:10.1002/anie.202518500)
Supplement: Supplementary file 1 — Supporting Information [file ANIE-65-e18500-s001.docx]

**Supplementary Information**

**On the Nature of Improper Hydrogen Bonding in RCH_2_F and RCHF_2_ Motifs**

Bruno A. Piscelli,^1,2^ Michael Bühl,^2^ Rodrigo A. Cormanich,^1^* and David O’Hagan^2^*

*^1^UNICAMP, Universidade Estadual de Campinas, Instituto de Química, Rua Monteiro Lobato 270, 13083-862, Campinas, São Paulo, Brasil.*

*^2^EaStCHEM, School of Chemistry, University of St Andrews, St Andrews, Fife, KY16 9ST, UK.*

** Correspondence E. mails: do1@st-andrews.ac.uk*

*cormanich@unicamp.br*

Table of Contents

Materials and Methods………………………………………………………………………………………………………………………………………………………………..3

[Bent’s Rule and The Improper Hydrogen Bonds for Methane and Ethane Derivatives 4](#_Toc211353571)

[Hydrogen Bonding in -CH_2_F and -CHF_2_ Motifs *vs* -OH (Methanol) 6](#_Toc211353572)

[Comparison of Atomic Charges Obtained at Different Methods for Difluoroethane 7](#_Toc211353573)

[Figure S3. Calculated scans varying C-H∙∙∙OH_2_ distance in increments of 0.1 Å for methane derivatives. NPA charges on interacting H, C and F atoms, C-H bond length and BSSE-corrected interaction energies. Calculations at the B3LYP-D3/def2-TZVP theoretical level. 9](#_Toc211353574)

[Figure S4. Calculated scans varying C-H∙∙∙OH_2_ distance in increments of 0.1 Å for ethane derivatives. NPA charges on interacting H, C and F atoms, C-H bond length and BSSE-corrected interaction energies. Calculations at the B3LYP-D3/def2-TZVP theoretical level. 10](#_Toc211353575)

[Table S2. DLPNO-CCSD(T)/aug-cc-pVQZ/LED//B3LYP-D3/def-QZVP terms for methane and ethane derivatives interacting with H_2_O. 11](#_Toc211353576)

[Global Minimum Structures for Selected Complexes with Water 12](#_Toc211353577)

[Table S3. NBO steric interactions (in kcal mol^-1^) for methane and ethane derivatives global minimum complexes with Cl^-^ obtained at the B3LYP-D3/def2-QZVP(D) theoretical level. 13](#_Toc211353578)

[Solvation Effect on Chloride-Binding Properties of Fluorinated Alicycles 14](#_Toc211353579)

[References 15](#_Toc211353580)

# Materials and methods

***Materials and Methods***

Relaxed potential energy curves for methane and ethane derivatives were obtained by scanning the C-H∙∙∙Cl^-^ (or OH_2_) distance in increments of 0.1 Å from 1.4 to 3.5 Å constraining the C-H∙∙∙Cl^-^ (or OH_2_) angle to 180^o^, using the GGA-hybrid functional B3LYP ^[1]^ in conjunction with Grimme’s D3 empirical correction^[2]^ and Alrich’s triple-ζ def2-TZVP basis set,^[3]^ using Gaussian16 Rev. C.01 software.^[4]^ To account for the polarizable nature of the chloride anion, additional diffuse functions were added to this atom (def2-TZVPD).^[5]^ NBO analysis^[6]^ was carried out at each point in the potential energy curve at the same theoretical level using NBO 7.0 as implemented in Gaussian16. The geometries of minima found in each curve were re-optimized with the quadruple-ζ def2-QZVP ^[7]^ basis set (def2-QZVPD ^[5]^ on Cl^-^), constraining the C-H∙∙∙Cl^-^ (or OH_2_) distance and angle. High-level electronic complexation energies over the quadruple-ζ quality geometries were obtained employing the Domain-Based Local Pair Natural Orbital (DLPNO) approximation to CCSD(T) ^[8]^ with Dunning’s aug-cc-pVQZ ^[9]^ quadruple-ζ basis set in ORCA 6.0.1 software ^[10]^ and BSSE-corrected following the Counter Poise method.^[11]^ The DLPNO-CCSD(T)/aug-cc-pVQZ wave function was then decomposed into chemically meaningful terms using the Local Energy Decomposition (LED) scheme ^[12]^ to DLPNO-CCSD(T) as implemented in ORCA 6.0.1.

The global minimum geometries for the complexes of methane and ethane derivatives with Cl^-^ were also studied. Initial geometries were obtained from a non-covalent conformational search employing the GFN2-xTB semi-empirical method ^[13]^ in CREST 2.12.2 software.^[14,15]^ The global minimum geometry was reoptimized in Gaussian16 at the B3LYP-D3/def2-QZVP(def2-QZVPD on Cl^-^) theoretical level (without constraints), showing no imaginary harmonic frequencies. Anharmonic frequencies were obtained following the Generalized Vibrational Perturbation Theory (GVPT2) method^[16]^ as implemented in Gaussian16 for the evaluation of red and blue shift upon hydrogen bonding. BSSE-corrected interaction energies were then obtained at the DLPNO-CCSD(T)/aug-cc-pVQZ level and decomposed using the Local Energy Decomposition (LED) scheme.^[17]^

For the monomeric systems, geometry optimization and NBO analysis were carried out at the B3LYP-D3/def2-QZVP level, with high-level electronic energies at the DLPNO-CCSD(T)/aug-cc-pVQZ level of theory.

The initial configuration of the alicyclic systems complexed to Cl^-^ were also assessed through a non-covalent conformational search employing GFN2-xTB and CREST software. The global minima structures were submitted to full geometry optimizations at B3LYP-D3/def2-TZVP(def2-TZVPD on Cl^-^) in Gaussian16. Harmonic frequencies were calculated at the same theory level, showing no imaginary frequencies. Reported interaction energies were BSSE-corrected following the Counter Poise method.

# Bent’s Rule and The Improper Hydrogen Bonds for Methane and Ethane Derivatives ^[18]^

An analysis on the basis of Bent’s rule^[18]^ reveals a clear trend in carbon rehybridization upon chloride complexation (Figure A1). As Cl⁻ approaches the C-H bond in methane, carbon increases the *s* character in the interacting C–H bond, shifting from ideal *sp*³ hybridization in the free molecule to *sp*^2.69^ in the complex. As predicted by Bent’s rule, this rehybridization helps to withdraw the incoming electron density from Cl⁻ to H and consequently increases the *p* character of the non-interacting C-H bonds (to *sp*^3.11^). A similar pattern is observed in mono-, di-, and trifluoromethane, where the *s* character of the interacting C-H bond increases progressively to *sp*^2.60^, *sp*^2.24^, and *sp*^1.72^, respectively, in all cases larger than in the non-complexed monomers. This essentially leads to an increase in electron density on the carbon center, allowing the F atoms to draw more charge and accumulate the density excess (as illustrated in Figure 3). To facilitate this, C allocates significant *p* character to the C-F bonds – reaching up to *sp*^4.83^ in fluoromethane. As more fluorines are added, the atoms compete for carbon’s available *p* character, leading to decreased hybridizations of *sp*^4.12^ and *sp*^3.70^ in the C-F bonds of difluoro- and trifluoromethane, respectively. Thus, in all cases, Cl⁻ complexation induces carbon rehybridization that enhances *s* character in the interacting C–H bond, which would typically favor bond strengthening and shortening. However, as discussed in the main text, in methane and trifluoromethane, strong *n*_Cl⁻_ → σ*_C–H_* hyperconjugative interactions override this effect, leading to red-shifted hydrogen bonds. In mono- and difluoromethane, where such charge transfer is considerably weaker, the rehybridization dominates, and the hydrogen bonds become blue-shifted.

A similar trend is observed for the ethane analogues, where the *s* character of the carbon atom in the interacting C–H bond increases relative to the unbound state, reaching *sp*^2.94^, *sp*^2.81^, *sp*^2.35^, and *sp*^2.60^ for ethane, fluoroethane, difluoroethane, and trifluoroethane, respectively. In these systems, the C–C bond remains largely unaffected in terms of hybridization. As in the methane series, the introduction of fluorine atoms provides an efficient pathway for delocalizing the excess electron density on the interacting hydrogen (see Figure 8), with C-F bond hybridizations reaching *sp*^5.08^ in fluoroethane and *sp*^4.36^ in difluoroethane. An exception arises in trifluoroethane, where the fluorine atoms are vicinal rather than geminal to the interacting hydrogen. In this case, the carbon bearing the interacting C-H bond cannot directly channel excess electron density to the fluorine atoms. Instead, the delocalization occurs through the vicinal carbon, leading to an increase in *p* character in the C–C bond (to *sp*^3.28^). As with methane derivatives, the balance between rehybridization and charge transfer governs the hydrogen bonding trends. In ethane, weak hyperconjugative *n*_Cl⁻_ → σ*_C–H_* interactions are nearly offset by carbon rehybridization, resulting in a small red shift. In fluoro- and difluoroethane, rehybridization dominates, compensating for weaker hyperconjugation and leading to blue-shifted hydrogen bonds. Finally, in trifluoroethane, although rehybridization of the interacting C-H bond is significant, it is outweighed by strong *n*_Cl⁻_ → σ*_C–H_* interactions, resulting in a pronounced red shift.

**Figure S1.** Hybridization on interacting carbon atoms on unbound and global-minimum complexed to Cl^-^ states of methane and ethane derivatives, obtained from NBO at the B3LYP-D3/def2-QZVP(D) theoretical level.

# Hydrogen Bonding in -CH_2_F and -CHF_2_ Motifs *vs* -OH (Methanol)

A similar analysis conducted for the methane as ethane derivatives was conducted for the classical hydrogen bond donor –OH group, using methanol as a representative compound. In this case, chloride complexation is primarily driven by interaction with the electropositive hydrogen atom, which carries a partial charge of +0.459e in unbound methanol. Upon approximation of Cl⁻ to the –OH group, the hydrogen atom becomes slightly more electropositive, accompanied by significant charge transfer to the oxygen atom. The interaction energy in this case (–16.0 kcal mol⁻¹) is considerably stronger than those observed for the methane and ethane analogues, as expected for a classical hydrogen bond donor like the hydroxyl group.

During complexation, strong electron donation from *n*_Cl⁻_ to the σ^*^_O-H_ orbital leads to a notable elongation of the O–H bond, from 0.959 to 0.993 Å, corresponding to a red shift of 257 cm⁻¹. Additionally, chloride binding induces a rehybridization at the oxygen atom, increasing the *s* character of the O–H bond from *sp*^3.84^ in the free state to *sp*^2.91^ upon complexation. Despite this increase, the bond retains a high degree of *p* character, remaining close to *sp*³ hybridization. The remaining large *p* character and strong *n*_Cl⁻_ → σ_O-H_ delocalization results in the characteristic red-shifted hydrogen bonds for the -OH···Cl⁻ interaction in methanol.

**Figure S2.** NPA-calculated atomic charges (+ve in blue, -ve in red) for the C, H and F atoms for methanol (B3LYP-D3/def2-QZVP); oxygen hybridization at unbound and complexed state [B3LYP-D3/def2-QZVP(D)]; calculated scan varying O-H···Cl⁻ distance in increments of 0.1 Å for methanol, with NPA charges on interacting H and O, C and O-H bond length and BSSE-corrected interaction energies [B3LYP-D3/def2-TZVP(D)]; Anharmonic C-H stretch frequencies differences between the non-complexed and complexed to Cl^-^ global minimum geometries of methanol and NBO hyperconjugative interaction between fragments [B3LYP-D3/def2-QZVP(D)], and interaction energies ($\Delta E$*_int_*) at the DLPNO-CCSD(T)/aug-cc-pVQZ.

# Comparison of Atomic Charges Obtained at Different Methods for Difluoroethane

The atomic charges of difluoroethane were calculated using B3LYP-D3^[1,2]^, M06-2X^[19]^ and the double-hybrid B2PLYP-D3BJ^[20,21]^ functionals, with Alrich’s def2-QZVP^[22]^ and Dunning’s aug-cc-pVQZ^[9]^ quadruple-ζ basis sets, at different classes of charge schemes (Table S1) using MultiWFN 3.8 ^[23]^:

i) WF-based: NPA^24^, Löwdin^25^ and Mulliken^[26-28]^;

ii) Real Space Partition: Atoms in Molecules (AIM)^[29,30]^, Hirshfeld-I^[31]^ and Voronoi deformation density (VDD)^[32]^;

iii) ESP-fitting: ChelpG^[33]^, Merz-Kollmann (MK)^[34]^ and Restrained ElectroStatic Potential (RESP)^[35]^;

iv) Others: Atomic Dipole-Corrected Hirshfeld (ADCH)^36^ and Charge Model 5 (CM5)^[37]^.

Importantly, all charge schemes and theoretical methods, except for the Löwdin scheme, consistently predict that C_α_ is significantly more electropositive than C_β_. Importantly, Löwdin charge scheme predicts positive atomic charges for the fluorine atoms in all theoretical methods tested, highlighting the weakness of this charge scheme for this system. Regarding the hydrogen atoms, H_β_ is generally found to be more electropositive than H_α_ across all methods, except for AIM, VDD, and CM5, which assign nearly identical atomic charges to both hydrogens. Overall, the consistent trends observed across multiple theoretical approaches and charge models provide a robust theoretical foundation for the conclusions presented in the main text in terms of NPA charges.

**Table S1.** Atomic charges for difluoroethane obtained at different theoretical methods and charge models.

|  | **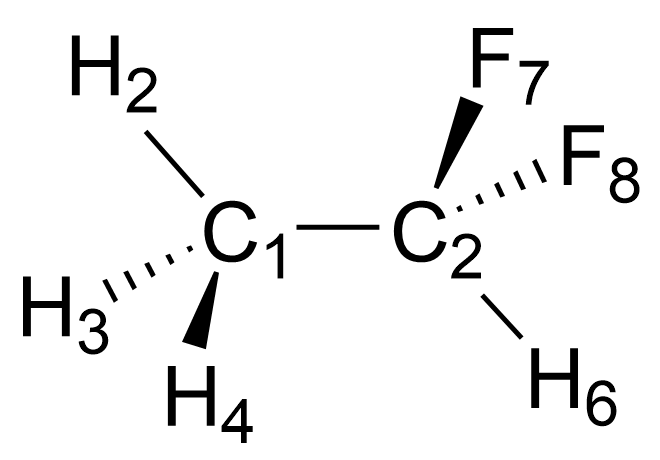** | | | | | | | | | | |
| --- | --- | --- | --- | --- | --- | --- | --- | --- | --- | --- | --- |
|  | **B3LYP-D3/def2-QZVP** | | | | | | | | | | |
|  | **WF-Based** | | | **Real-Space Partition** | | | **ESP-Fitting** | | | **Others** | |
| **Atom** | **NPA** | **Löwdin** | **Mulliken** | **AIM** | **Hirshfeld-I** | **Voronoi (VDD)** | **ChelpG** | **MK** | **RESP** | **ADCH** | **CM5** |
| **C1** | -0.667 | 0.226 | -0.117 | 0.110 | -0.626 | -0.083 | -0.378 | -0.478 | -0.474 | -0.275 | -0.234 |
| **H2** | 0.231 | -0.054 | 0.069 | 0.008 | 0.164 | 0.048 | 0.140 | 0.169 | 0.141 | 0.107 | 0.100 |
| **H3** | 0.225 | -0.055 | 0.061 | 0.003 | 0.160 | 0.047 | 0.105 | 0.131 | 0.141 | 0.117 | 0.099 |
| **H4** | 0.225 | -0.055 | 0.061 | 0.003 | 0.160 | 0.047 | 0.105 | 0.131 | 0.141 | 0.117 | 0.099 |
| **C5** | 0.572 | -0.668 | 0.486 | 1.177 | 0.633 | 0.179 | 0.571 | 0.581 | 0.606 | 0.173 | 0.153 |
| **H6** | 0.141 | -0.079 | 0.032 | 0.026 | 0.011 | 0.051 | 0.013 | 0.015 | -0.009 | 0.105 | 0.105 |
| **F7** | -0.363 | 0.342 | -0.296 | -0.662 | -0.251 | -0.145 | -0.278 | -0.275 | -0.273 | -0.172 | -0.161 |
| **F8** | -0.363 | 0.342 | -0.296 | -0.662 | -0.251 | -0.145 | -0.278 | -0.275 | -0.273 | -0.172 | -0.161 |
|  | **B2PLYP-D3/def2-QZVP** | | | | | | | | | | |
|  | **WF-Based** | | | **Real-Space Partition** | | | **ESP-Fitting** | | | **Others** | |
| **Atom** | **NPA** | **Löwdin** | **Mulliken** | **AIM** | **Hirshfeld-I** | **Voronoi (VDD)** | **ChelpG** | **MK** | **RESP** | **ADCH** | **CM5** |
| **C1** | -0.660 | 0.234 | -0.144 | 0.152 | -0.615 | -0.068 | -0.407 | -0.501 | -0.496 | -0.277 | -0.227 |
| **H2** | 0.228 | -0.055 | 0.080 | 0.000 | 0.160 | 0.044 | 0.149 | 0.176 | 0.146 | 0.109 | 0.100 |
| **H3** | 0.222 | -0.056 | 0.071 | -0.008 | 0.155 | 0.043 | 0.111 | 0.136 | 0.146 | 0.116 | 0.098 |
| **H4** | 0.222 | -0.056 | 0.071 | -0.008 | 0.155 | 0.043 | 0.111 | 0.136 | 0.146 | 0.116 | 0.098 |
| **C5** | 0.574 | -0.650 | 0.501 | 1.262 | 0.712 | 0.212 | 0.608 | 0.620 | 0.648 | 0.198 | 0.180 |
| **H6** | 0.136 | -0.076 | 0.043 | 0.023 | 0.003 | 0.053 | 0.014 | 0.015 | -0.012 | 0.108 | 0.108 |
| **F7** | -0.360 | 0.329 | -0.311 | -0.709 | -0.285 | -0.163 | -0.293 | -0.291 | -0.289 | -0.186 | -0.179 |
| **F8** | -0.360 | 0.329 | -0.311 | -0.709 | -0.285 | -0.163 | -0.293 | -0.291 | -0.289 | -0.186 | -0.179 |
|  | **M06-2X/def2-QZVP** | | | | | | | | | | |
|  | **WF-Based** | | | **WF-Based** | | | **WF-Based** | | | **WF-Based** | |
| **Atom** | **NPA** | **Löwdin** | **Mulliken** | **AIM** | **Hirshfeld-I** | **Voronoi (VDD)** | **ChelpG** | **MK** | **RESP** | **ADCH** | **CM5** |
| **C1** | -0.675 | 0.225 | -0.621 | 0.111 | -0.642 | -0.081 | -0.441 | -0.534 | -0.530 | -0.288 | -0.236 |
| **H2** | 0.232 | -0.052 | 0.216 | 0.010 | 0.168 | 0.047 | 0.158 | 0.185 | 0.157 | 0.111 | 0.102 |
| **H3** | 0.227 | -0.054 | 0.218 | -0.001 | 0.164 | 0.046 | 0.122 | 0.147 | 0.157 | 0.120 | 0.100 |
| **H4** | 0.227 | -0.054 | 0.218 | -0.001 | 0.164 | 0.046 | 0.122 | 0.147 | 0.157 | 0.120 | 0.100 |
| **C5** | 0.587 | -0.705 | 0.323 | 1.243 | 0.650 | 0.185 | 0.568 | 0.579 | 0.604 | 0.169 | 0.156 |
| **H6** | 0.140 | -0.075 | 0.141 | 0.020 | 0.013 | 0.054 | 0.021 | 0.022 | -0.002 | 0.111 | 0.108 |
| **F7** | -0.369 | 0.357 | -0.248 | -0.690 | -0.258 | -0.148 | -0.276 | -0.273 | -0.271 | -0.171 | -0.165 |
| **F8** | -0.369 | 0.357 | -0.248 | -0.690 | -0.258 | -0.148 | -0.275 | -0.273 | -0.271 | -0.171 | -0.165 |

**Table S1.** Continuation.

|  | **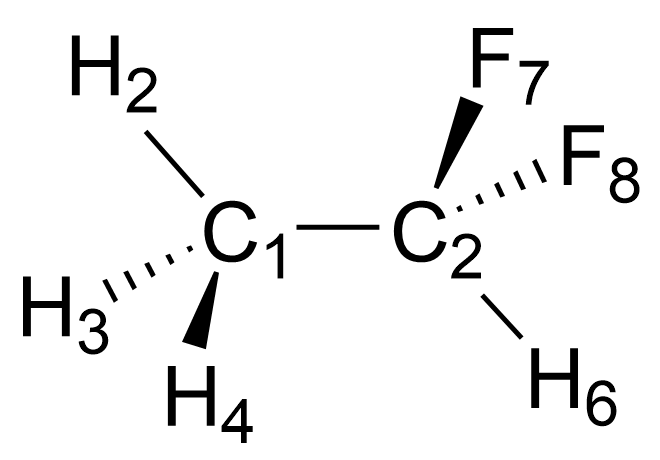** | | | | | | | | | | |
| --- | --- | --- | --- | --- | --- | --- | --- | --- | --- | --- | --- |
|  | **B3LYP-D3/aug-cc-pVQZ** | | | | | | | | | | |
|  | **WF-Based** | | | **Real-Space Partition** | | | **ESP-Fitting** | | | **Others** | |
| **Atom** | **NPA** | **Löwdin** | **Mulliken** | **AIM** | **Hirshfeld-I** | **Voronoi (VDD)** | **ChelpG** | **MK** | **RESP** | **ADCH** | **CM5** |
| **C1** | -0.666 | 0.828 | -0.929 | 0.126 | -0.628 | -0.082 | -0.377 | -0.477 | -0.473 | -0.275 | -0.234 |
| **H2** | 0.230 | -0.313 | 0.275 | 0.007 | 0.164 | 0.048 | 0.140 | 0.169 | 0.141 | 0.106 | 0.100 |
| **H3** | 0.225 | -0.305 | 0.316 | -0.002 | 0.160 | 0.047 | 0.104 | 0.131 | 0.141 | 0.117 | 0.099 |
| **H4** | 0.225 | -0.305 | 0.316 | -0.002 | 0.160 | 0.047 | 0.104 | 0.131 | 0.141 | 0.117 | 0.099 |
| **C5** | 0.551 | -1.069 | 0.858 | 1.177 | 0.633 | 0.180 | 0.574 | 0.585 | 0.610 | 0.173 | 0.152 |
| **H6** | 0.133 | -0.411 | 0.365 | 0.022 | 0.011 | 0.051 | 0.012 | 0.014 | -0.011 | 0.105 | 0.105 |
| **F7** | -0.349 | 0.787 | -0.600 | -0.663 | -0.251 | -0.146 | -0.279 | -0.276 | -0.274 | -0.172 | -0.161 |
| **F8** | -0.349 | 0.787 | -0.600 | -0.663 | -0.251 | -0.146 | -0.279 | -0.276 | -0.274 | -0.172 | -0.161 |
|  | **B2PLYP-D3/aug-cc-pVQZ** | | | | | | | | | | |
|  | **WF-Based** | | | **Real-Space Partition** | | | **ESP-Fitting** | | | **Others** | |
| **Atom** | **NPA** | **Löwdin** | **Mulliken** | **AIM** | **Hirshfeld-I** | **Voronoi (VDD)** | **ChelpG** | **MK** | **RESP** | **ADCH** | **CM5** |
| **C1** | -0.659 | 0.840 | -0.945 | 0.169 | -0.617 | -0.067 | -0.406 | -0.500 | -0.497 | -0.276 | -0.227 |
| **H2** | 0.227 | -0.313 | 0.278 | -0.002 | 0.161 | 0.044 | 0.149 | 0.176 | 0.146 | 0.109 | 0.100 |
| **H3** | 0.221 | -0.306 | 0.315 | -0.014 | 0.155 | 0.043 | 0.111 | 0.136 | 0.146 | 0.117 | 0.098 |
| **H4** | 0.221 | -0.306 | 0.315 | -0.014 | 0.155 | 0.043 | 0.111 | 0.136 | 0.146 | 0.117 | 0.098 |
| **C5** | 0.553 | -1.063 | 0.911 | 1.263 | 0.712 | 0.212 | 0.608 | 0.621 | 0.648 | 0.198 | 0.180 |
| **H6** | 0.129 | -0.409 | 0.373 | 0.020 | 0.004 | 0.053 | 0.014 | 0.015 | -0.012 | 0.108 | 0.108 |
| **F7** | -0.346 | 0.778 | -0.623 | -0.709 | -0.285 | -0.164 | -0.293 | -0.291 | -0.289 | -0.186 | -0.179 |
| **F8** | -0.346 | 0.778 | -0.623 | -0.709 | -0.285 | -0.164 | -0.293 | -0.291 | -0.289 | -0.186 | -0.179 |
|  | **M06-2X/aug-cc-pVQZ** | | | | | | | | | | |
|  | **WF-Based** | | | **Real-Space Partition** | | | **ESP-Fitting** | | | **Others** | |
| **Atom** | **NPA** | **Löwdin** | **Mulliken** | **AIM** | **Hirshfeld-I** | **Voronoi (VDD)** | **ChelpG** | **MK** | **RESP** | **ADCH** | **CM5** |
| **C1** | -0.672 | 0.834 | -1.728 | 0.158 | -0.641 | -0.080 | -0.432 | -0.526 | -0.523 | -0.286 | -0.236 |
| **H2** | 0.231 | -0.313 | 0.567 | -0.011 | 0.168 | 0.047 | 0.156 | 0.183 | 0.155 | 0.110 | 0.101 |
| **H3** | 0.225 | -0.306 | 0.554 | -0.014 | 0.164 | 0.045 | 0.121 | 0.146 | 0.155 | 0.120 | 0.100 |
| **H4** | 0.225 | -0.306 | 0.554 | -0.014 | 0.164 | 0.045 | 0.120 | 0.146 | 0.155 | 0.120 | 0.100 |
| **C5** | 0.564 | -1.108 | 0.395 | 1.248 | 0.649 | 0.185 | 0.560 | 0.571 | 0.596 | 0.167 | 0.155 |
| **H6** | 0.132 | -0.411 | 0.499 | 0.012 | 0.012 | 0.053 | 0.022 | 0.023 | -0.001 | 0.110 | 0.107 |
| **F7** | -0.353 | 0.805 | -0.421 | -0.689 | -0.257 | -0.148 | -0.273 | -0.271 | -0.269 | -0.170 | -0.164 |
| **F8** | -0.353 | 0.805 | -0.421 | -0.689 | -0.257 | -0.148 | -0.273 | -0.271 | -0.269 | -0.170 | -0.164 |

# Figure S3. Calculated scans varying C-H∙∙∙OH_2_ distance in increments of 0.1 Å for methane derivatives. NPA charges on interacting H, C and F atoms, C-H bond length and BSSE-corrected interaction energies. Calculations at the B3LYP-D3/def2-TZVP theoretical level.

# Figure S4. Calculated scans varying C-H∙∙∙OH_2_ distance in increments of 0.1 Å for ethane derivatives. NPA charges on interacting H, C and F atoms, C-H bond length and BSSE-corrected interaction energies. Calculations at the B3LYP-D3/def2-TZVP theoretical level.

# Table S2. DLPNO-CCSD(T)/aug-cc-pVQZ/LED//B3LYP-D3/def-QZVP terms for methane and ethane derivatives interacting with H_2_O.

|  | **Methane** | **Fluoromethane** | **Difluoromethane** | **Trifluoromethane** |
| --- | --- | --- | --- | --- |
| $\boldsymbol{\Delta E}_{\boldsymbol{geo-prep}}$ | 0.01 | 0.05 | 0.08 | 0.17 |
| $\boldsymbol{\Delta}\boldsymbol{E}_{\boldsymbol{el-prep}}^{\boldsymbol{HF}}$ | 4.78 | 5.98 | 8.96 | 11.14 |
| $\boldsymbol{E}_{\boldsymbol{elstat}}^{\boldsymbol{HF}}$ | -3.73 | -5.85 | -9.31 | -12.28 |
| $\boldsymbol{E}_{\boldsymbol{exch}}^{\boldsymbol{HF}}$ | -0.86 | -1.07 | -1.54 | -1.84 |
| $\boldsymbol{\Delta E}_{\boldsymbol{non-disp}}^{\boldsymbol{C}}$ | 0.01 | 0.13 | 0.23 | 0.42 |
| $\boldsymbol{\Delta E}_{\boldsymbol{disp}}^{\boldsymbol{C}}$ | -0.73 | -0.85 | -0.97 | -1.13 |
| $\boldsymbol{\Delta E}_{\boldsymbol{int}}^{\boldsymbol{C-(T)}}$ | -0.12 | -0.09 | -0.08 | -0.07 |
| $\boldsymbol{E}_{\boldsymbol{BSSE}}$ | 0.05 | 0.11 | 0.14 | 0.18 |
| $\boldsymbol{\Delta}\boldsymbol{E}_{\boldsymbol{int}}$ | -0.59 | -1.60 | -2.50 | -3.42 |
|  | **Ethane** | **Fluoroethane-1** | **Fluoroethane-2** | **Fluoroethane-3** |
| $\boldsymbol{\Delta E}_{\boldsymbol{geo-prep}}$ | 0.01 | 0.05 | 0.05 | 0.03 |
| $\boldsymbol{\Delta}\boldsymbol{E}_{\boldsymbol{el-prep}}^{\boldsymbol{HF}}$ | 4.85 | 5.15 | 5.73 | 5.05 |
| $\boldsymbol{E}_{\boldsymbol{elstat}}^{\boldsymbol{HF}}$ | -3.69 | -5.10 | -5.17 | -4.80 |
| $\boldsymbol{E}_{\boldsymbol{exch}}^{\boldsymbol{HF}}$ | -0.88 | -0.95 | -1.07 | -0.91 |
| $\boldsymbol{\Delta E}_{\boldsymbol{non-disp}}^{\boldsymbol{C}}$ | -0.02 | 0.10 | 0.05 | 0.08 |
| $\boldsymbol{\Delta E}_{\boldsymbol{disp}}^{\boldsymbol{C}}$ | -0.82 | -0.82 | -1.00 | -0.82 |
| $\boldsymbol{\Delta E}_{\boldsymbol{int}}^{\boldsymbol{C-(T)}}$ | -0.13 | -0.09 | -0.13 | -0.11 |
| $\boldsymbol{E}_{\boldsymbol{BSSE}}$ | 0.09 | 0.11 | 0.11 | 0.10 |
| $\boldsymbol{\Delta}\boldsymbol{E}_{\boldsymbol{int}}$ | -0.59 | -1.54 | -1.42 | -1.38 |
|  | **Difluoroethane-1** | **Difluoroethane-2** | **Difluoroethane-3** | **Trifluoroethane** |
| $\boldsymbol{\Delta E}_{\boldsymbol{geo-prep}}$ | 0.10 | 0.05 | 0.06 | 0.07 |
| $\boldsymbol{\Delta}\boldsymbol{E}_{\boldsymbol{el-prep}}^{\boldsymbol{HF}}$ | 9.47 | 7.79 | 6.35 | 9.41 |
| $\boldsymbol{E}_{\boldsymbol{elstat}}^{\boldsymbol{HF}}$ | -9.43 | -7.22 | -6.36 | -9.05 |
| $\boldsymbol{E}_{\boldsymbol{exch}}^{\boldsymbol{HF}}$ | -1.64 | -1.44 | -1.14 | -1.66 |
| $\boldsymbol{\Delta E}_{\boldsymbol{non-disp}}^{\boldsymbol{C}}$ | 0.14 | 0.05 | 0.12 | 0.14 |
| $\boldsymbol{\Delta E}_{\boldsymbol{disp}}^{\boldsymbol{C}}$ | -1.02 | -1.04 | -0.96 | -1.15 |
| $\boldsymbol{\Delta E}_{\boldsymbol{int}}^{\boldsymbol{C-(T)}}$ | -0.10 | -0.14 | -0.11 | -0.13 |
| $\boldsymbol{E}_{\boldsymbol{BSSE}}$ | 0.16 | 0.15 | 0.11 | 0.15 |
| $\boldsymbol{\Delta}\boldsymbol{E}_{\boldsymbol{int}}$ | -2.32 | -1.82 | -1.92 | -2.05 |

# Global Minimum Structures for Selected Complexes with Water

**Figure S5.** Global minimum geometries of mono- and difluoro methane (and ethane) derivatives complexed to water, NPA atomic charges (+v_e_ in blue, -v_e_ in red) and selected distances intermolecular distances obtained at the B3LYP-D3/def2-QZVP theoretical level. Interaction energies (∆*E*_int_) in kcal mol^-1^ at DLPNO-CCSD(T)/aug-cc-pVQZ//B3LYP-D3/def2-QZVP, in kcal mol^-1^.

# Table S3. NBO steric interactions (in kcal mol^-1^) for methane and ethane derivatives global minimum complexes with Cl^-^ obtained at the B3LYP-D3/def2-QZVP(D) theoretical level.

|  | **Methane** | **Fluoromethane** | **Difluoromethane** | **Trifluoromethane** |
| --- | --- | --- | --- | --- |
| **Total Inter-Fragment Steric Interaction** | 4.12 | 5.53 | 6.52 | 10.94 |
| ***n*_Cl-_ ↔ σ_CH_** | 3.97 | *3x* 1.87 | *2x* 3.23 | 10.71 |
|  | **Ethane** | **Fluoroethane** | **Difluoroethane** | **Trifluoroethane** |
| **Total Inter-Fragment Steric Interaction** | 4.99 | 7.66 | 8.66 | 8.16 |
| ***n*_Cl-_ ↔ σ_CH_** | 3.86 | *2x* 1.57 | 4.98 | 7.40 |

# Solvation Effect on Chloride-Binding Properties of Fluorinated Alicycles

**Figure S6.** Accounting for acetonitrile. Chloride ion complexation energies (kcal mol^-1^) and NPA atomic charges (+v_e_ in blue, -v_e_ in red) calculated at the SMD(MeCN)-B3LYP-D3/def2-TZVP(D) theory level for alicyclic rings **1**-**14** compared to urea.

# References

[1] P. J. Stephens, F. J. Devlin, C. F. Chabalowski, M. J. Frisch Ab initio calculation of vibrational absorption and circular dichroism spectra using density functional force fields. *J. Phys. Chem.*, **1994**, *98*, 11623-11627.

[2] S. Grimme, J. Antony, S. Ehrlich, H. Krieg, A consistent and accurate ab initio parametrization of density functional dispersion correction (DFT-D) for the 94 elements H-Pu. *J. Phys. Chem.*, **2010**, *132*, 154104-154123.

[3] F. Weigend, R. Ahlrichs, Balanced basis sets of split valence, triple zeta valence and

quadruple zeta valence quality for H to Rn: Design and assessment of accuracy. *Phys. Chem. Chem. Phys.* **2005**, *7*, 3297-3305.

[4] M. J. Frisch, G. W. Trucks, H. B. Schlegel, G. E. Scuseria, M. A. Robb, J. R. Cheeseman, G. Scalmani, V. Barone, G. A. Petersson, H. Nakatsuji, X. Li, M. Caricato, A. V. Marenich, J. Bloino, B. G. Janesko, R. Gomperts, B. Mennucci, H. P. Hratchian, J. V. Ortiz, A. F. Izmaylov, J. L. Sonnenberg, D. Williams-Young, F. Ding, F. Lipparini, F. Egidi, J. Goings, B. Peng, A. Petrone, T. Henderson, D. Ranasinghe, V. G. Zakrzewski, J. Gao, N. Rega, G. Zheng, W. Liang, M. Hada, M. Ehara, K. Toyota, R. Fukuda, J. Hasegawa, M. Ishida, T. Nakajima, Y. Honda, O. Kitao, H. Nakai, T. Vreven, K. Throssell, J. A. Montgomery, Jr., J. E. Peralta, F. Ogliaro, M. J. Bearpark, J. J. Heyd, E. N. Brothers, K. N. Kudin, V. N. Staroverov, T. A. Keith, R. Kobayashi, J. Normand, K. Raghavachari, A. P. Rendell, J. C. Burant, S. S. Iyengar, J. Tomasi, M. Cossi, J. M. Millam, M. Klene, C. Adamo, R. Cammi, J. W. Ochterski, R. L. Martin, K. Morokuma, O. Farkas, J. B. Foresman, and D. J. Fox,. Gaussian 16, Revision C.01; Gaussian, Inc.: Wallingford, CT, 2016.

[5] D. Rappoport, F. Furche, Property-optimized Gaussian basis sets for molecular response calculations. *J. Chem. Phys.*, **2010**, *133*, 134105-134116.

[6] E. D. Glendening, J. K. Badenhoop, A. E. Reed J. E. Carpenter, J. A. Bohmann, C. M. Morales, P. Karafiloglou, C. R. Landis, F. Weinhold, NBO 7.0, Theoretical Chemistry Institute, University of Wisconsin, Madison, **2018**.

[7] F. Weigend, F. Furche, R. Ahlrichs, Gaussian basis sets of quadruple zeta valence quality for atoms H–Kr. *J. Chem. Phys*., **2003**, *119*, 12753-12762.

[8] C. Riplinger, F. Neese, An efficient and near linear scaling pair natural orbital based local coupled cluster method. *J. Chem. Phys.*, **2013**, *138*, 034106–034118.

[9] R. A. Kendall, T. H. Dunning Jr., R. J. Harrison, Electron affinities of the first‐row atoms revisited. Systematic basis sets and wave functions. *J. Chem. Phys.*, **1992**, *96*, 6796-6806.

[10] F. Neese, Software update: The ORCA program system-version 5.0. *Wires Comput. Molec. Sci.*, **2022**, *12*, e1606.

[11] S. F. Boys, F. Bernardi, F. The calculation of small molecular interactions by the differences of separate total energies. Some procedures with reduced errors. *Mol. Phys.*, **1970**, *19*, 553-566.

[12] W. B. Schneider, G. Bistoni, M. Sparta, M. Saitow, C. Riplinger, A. A. Auer, F. Neese, Decomposition of intermolecular interaction energies within the local pair natural orbital coupled cluster framework. *J. Theo. Comp. Chem.*, **2016**, *12*, 4778-4792.

[13] C. Bannwarth, S. Ehlert, S. Grimme, GFN2-xTB—An accurate and broadly parametrized self-consistent tight-binding quantum chemical method with multipole electrostatics and density-dependent dispersion contributions. *J. Chem. Theory Comput.*, **2019**, *15*, 1652–1671.

[14] P. Pracht, F. Bohle, S. Grimme, Automated exploration of the low-energy chemical space with fast quantum chemical methods. *Phys. Chem. Chem. Phys.,* **2020**, *22*, 7169–7192.

[15] S. Grimme, Exploration of chemical compound, conformer, and reaction space with meta-dynamics simulations based on tight-binding quantum chemical calculations. *J. Chem. Theory. Comput.*, **2019**, *15*, 2847–2862.

[16] V. Barone, Anharmonic vibrational properties by a fully automated second-order perturbative approach. *J. Chem. Phys.*, **2005**, *122*, 14108-14118.

[17] A. Altun, F. Neese, G. Bistoni, Local energy decomposition analysis of hydrogen-bonded dimers within a domain-based pair natural orbital coupled cluster study. *Beilstein J. Org. Chem.*, **2018**, *14*, 919 – 929.

[18] H. A. Bent, An appraisal of valence-bond structures and hybridization in compounds of the first-row elements. *Chem. Rev.*, **1961**, *61*, 275–311.

[19] Y. Zhao, D. G. Truhlar, The M06 Suite of Density Functionals for Main Group Thermochemistry, Thermochemical Kinetics, Noncovalent Interactions, Excited States, and Transition Elements: Two New Functionals and Systematic Testing of Four M06-Class Functionals and 12 Other Functionals. *Theor Chem Acc* **2008**, *120* (1), 215–241.

[20] S. Grimme, Semiempirical Hybrid Density Functional with Perturbative Second-Order Correlation. *J Chem Phys* **2006**, *124*, 034108.

[21] E. R. Johnson, A. D. Becke, A Post-Hartree-Fock Model of Intermolecular Interactions: Inclusion of Higher-Order Corrections. *J Chem Phys* **2006**, *124*, 174104.

[22] F. Weigend, F. Furche, R. Ahlrichs, Gaussian Basis Sets of Quadruple Zeta Valence Quality for Atoms H–Kr. *J Chem Phys* **2003**, *119*, 12753–12762.

[23] T. Lu, A Comprehensive Electron Wavefunction Analysis Toolbox for Chemists, Multiwfn. *J Chem Phys* **2024**, *161*, 082503.

[24] A. E. Reed, R. B. Weinstock, F. Weinhold, Natural Population Analysis. *J Chem Phys* **1985**, *83*, 735–746.

[25] L. Ch. Cusachs, P. Politzer, On the Problem of Defining the Charge on an Atom in a Molecule. *Chem Phys Lett* **1968**, *1*, 529–531.

[26] R. S. Mulliken, Electronic Population Analysis on LCAO–MO Molecular Wave Functions. I. *J Chem Phys* **1955**, *23*, 1833–1840.

[27] R. S. Mulliken, Electronic Population Analysis on LCAO–MO Molecular Wave Functions. II. Overlap Populations, Bond Orders, and Covalent Bond Energies. *J Chem Phys* **1955**, *23*, 1841–1846.

[28] R. S. Mulliken, Electronic Population Analysis on LCAO‐MO Molecular Wave Functions. III. Effects of Hybridization on Overlap and Gross AO Populations. *J Chem Phys* **1955**, *23*, 2338–2342.

[29] R. F. W. Bader, P. M. Beddall, Virial Field Relationship for Molecular Charge Distributions and the Spatial Partitioning of Molecular Properties. *J Chem Phys* **1972**, *56*, 3320–3329.

[30] R. F. W. Bader, *Atoms in Molecules*; Oxford University Press, Oxford, **1990**.

[31] P. Bultinck, C. Van Alsenoy, P. W. Ayers, R. Carbó-Dorca, Critical Analysis and Extension of the Hirshfeld Atoms in Molecules. *J Chem Phys* **2007**, *126*, 144111.

[32] C. Fonseca-Guerra, J. W. Handgraaf, E. J. Baerends, F. M. Bickelhaupt, Voronoi Deformation Density (VDD) Charges: Assessment of the Mulliken, Bader, Hirshfeld, Weinhold, and VDD Methods for Charge Analysis. *J Comput Chem* **2004**, *25*, 189–210.

[33] C. M. Breneman, K. B. Wiberg, Determining Atom-Centered Monopoles from Molecular Electrostatic Potentials. The Need for High Sampling Density in Formamide Conformational Analysis. *J Comput Chem* **1990**, *11*, 361–373.

[34] B. H. Besler, K. M. Merz Jr., P. A. Kollman, Atomic Charges Derived from Semiempirical Methods. *J Comput Chem* **1990**, *11*, 431–439.

[35] C. I. Bayly, P. Cieplak, W. Cornell, P. A. Kollman, A Well-Behaved Electrostatic Potential Based Method Using Charge Restraints for Deriving Atomic Charges: The RESP Model. *J Phys Chem* **1993**, *97*, 10269–10280.

[36] T. Lu, F. Chen, Atomic Dipole Moment Corrected Hirshfeld Population Method. *J Theor Comput Chem* **2012**, *11*, 163–183.

[37] A. V. Marenich, S. V. Jerome, C. J. Cramer, D. G. Truhlar, Charge Model 5: An Extension of Hirshfeld Population Analysis for the Accurate Description of Molecular Interactions in Gaseous and Condensed Phases. *J Chem Theory Comput* **2012**, *8*, 527–541.
